# Multimodal magnetic resonance imaging-based clustering identifies two imaging-defined brain phenotypes in De novo Parkinson's disease

**DOI:** 10.1016/j.nicl.2026.104021

**Published:** 2026-06-09

**Authors:** Esraa Arabi, Yuan Tian, Minwei Zhu, Wenpeng Gao, Yili Fu

**Affiliations:** a School of Life Science and Technology, Harbin Institute of Technology, Harbin, China; b State Key Laboratory of Robotics and System, Harbin Institute of Technology, Harbin, China; c Department of Neurosurgery, First Affiliated Hospital of Harbin Medical University, Nangang District, Harbin, China; d Department of Radiology, First Affiliated Hospital of Harbin Medical University, Nangang District, Harbin, China; e Department of Electrical Engineering, Benha faculty of engineering, Benha University, Benha, Egypt

**Keywords:** Parkinson's subtyping, Hierarchical clustering, Multimodal MRI, Normative modeling, Diffusion tensor imaging

## Abstract

Parkinson's disease (PD) exhibits marked clinical and neurobiological heterogeneity, complicating its prognosis and therapeutic development. We aimed to identify imaging-defined PD subtypes by integrating diffusion and structural MRI features, using a data-driven clustering approach. Baseline neuroimaging data from 103 de novo PD participants and 40 normal controls (NC) were obtained from the Parkinson's Progression Markers Initiative (PPMI). 209 imaging features were extracted, including fractional anisotropy (FA) and mean diffusivity (MD) from diffusion MRI and cortical and subcortical volumes from structural MRI. Imaging features were adjusted for demographic, anatomical, and scanner-related confounders using a normative, interaction-aware regression model. Hierarchical clustering with Ward's linkage was applied to the principal components of the adjusted features. The optimal cluster number was determined using Silhouette and Calinski–Harabasz indices, and cluster stability was evaluated by cross-algorithm validation and bootstrapping. Two imaging-defined PD subtypes were identified. Subtype A exhibited widespread elevations in FA and reductions in MD across multiple white matter tracts, with no significant volumetric atrophy, higher Symbol Digit Modalities Test performance, and an earlier age at onset. Subtype B showed widespread MD increases, regionally specific FA reductions, trend-level cortical volumetric reductions, ventricular enlargement, and lower cross-sectional cognitive performance. Over the 48-month follow-up, a linear mixed-effects model demonstrated divergent cognitive trajectories: Subtype A showed stable cognitive scores, whereas Subtype B showed significant cognitive decline. The two subtypes showed divergent cognitive trajectories in the longitudinal analysis. These findings identify two imaging-defined structural phenotypes in early de novo PD with divergent cognitive trajectories, providing a multimodal imaging framework for understanding PD heterogeneity at the earliest stages of the disease.

## Highlights

• Unsupervised clustering of 103 de novo PD participants identifies two reproducible imaging phenotypes.
• Normative age-interaction modeling isolates disease-specific aging effects.
• Diffusion metrics detect heterogeneity beyond volumetric atrophy.
• FA-preserved subtype shows widespread white matter anisotropy elevation.
• Diffuse-degeneration subtype shows faster cognitive decline over 48-month follow-up.

## 1 Introduction

### 1.1 Background

Despite sharing the same diagnosis, patients with Parkinson's disease (PD) have significantly distinct clinical trajectories, challenging the validity of a one-size-fits-all disease model(Deng et al., 2025). While conventionally defined by its cardinal motor features(Stebbins et al., 2013), PD exhibits remarkable variability in symptom presentation, progression rates, and treatment response(Berg et al., 2021), complicating clinical management, prognosis prediction, and therapeutic trial design(Severson et al., 2021).

The need to delineate clinically meaningful PD subtypes has evolved from hypothesis-driven approaches to sophisticated data-driven approaches that leverage multidimensional clinical and biological data(Albrecht et al., 2022; Deng et al., 2025). Neuroimaging has emerged as a critical tool for elucidating the neurobiological basis of PD heterogeneity. Structural magnetic resonance imaging (sMRI) is a valuable non-invasive tool for assessing macrostructural brain degeneration in PD, providing insights into the clinical presentations and PD prognoses. Previous studies used cortical thickness to reveal distinct PD patterns associated with differential neuropsychological, and motor characteristics, cognitive profiles and age of disease onset. Structural magnetic resonance imaging (sMRI) has been widely used to characterize macrostructural brain degeneration, revealing distinct cortical and subcortical atrophy patterns associated with differential clinical profiles in de novo and non-demented PD cohorts(Ibarretxe-Bilbao et al., 2009; Pan et al., 2024; Uribe et al., 2018; Uribe et al., 2016). However, macrostructural alterations may represent relatively late changes in neurodegeneration and may not fully capture early microstructural pathology.

Diffusion MRI (dMRI) serves as a sensitive probe of microstructural white matter integrity(Camacho et al., 2024; Zhang and Burock, 2020). In de novo and early-stage PD, DTI metrics such as Fractional Anisotropy (FA) and Mean Diffusivity (MD) have demonstrated the ability to detect axonal degeneration and neuroinflammatory changes that may precede volumetric atrophy(Atkinson-Clement et al., 2017; Hattori et al., 2012; Shih et al., 2023). While FA has been extensively studied, MD captures isotropic diffusion changes that may be particularly informative in extremely early stages of the disease and regions with complex fiber architecture. Although DTI has been extensively utilized to study white matter changes in PD, its application alone for explicit patient subtyping remains underdeveloped. This may be attributed to the fact that when DTI is utilized as a standalone modality for clustering or classification, it often underperforms(Panahi and Hosseini, 2024). Recent studies increasingly treat DTI as complementary to other modalities rather than as a primary clustering tool(Ni et al., 2025), such as the integration between sMRI and DTI, which offers a comprehensive view of PD-related neurodegeneration across macrostructural and microstructural scales(Inguanzo et al., 2021).

Although multimodal imaging research has expanded significantly, the specific integration of sMRI and DTI within an unsupervised, data-driven clustering framework remains unexplored. This limitation is compounded by the inadequate adjustment of biological features before applying a clustering algorithm for confounders, such as age and sex, which may confound subtype identification as proxies for demographic factors rather than true biological variances, thereby limiting the biological interpretability and clinical relevance.

### 1.2 Objectives

To bridge these gaps, the current study employed a multimodal neuroimaging framework that integrated whole-brain sMRI and DTI features for PD subtyping. Specifically, we combined cortical and subcortical volumetric measures derived from the Harvard-Oxford atlas with regional and tract-based FA and MD metrics from the JHU white matter atlases, encompassing 209 features to provide a whole-brain perspective. Emphasizing both FA and MD addresses the overreliance on FA, enabling a more nuanced assessment of white matter integrity. In addition, a normative, interaction-aware adjustment framework was applied to account for age, sex, education, scanner model, and total intracranial volume (TIV)-related effects prior to clustering, ensuring that the identified subtypes reflected disease-specific biological variation. Focusing on de novo PD, this study aimed to identify imaging-defined PD subtypes using a hierarchical clustering approach, thereby enhancing our understanding of PD heterogeneity. In addition, we aimed to demonstrate the different clinical profiles of early-stage PD patients associated with varying biological patterns.

## 2 Method

### 2.1 Data acquisition

#### 2.1.1 Image acquisition

A total of 103 patients with de novo PD and 40 NC were included in this study from the Parkinson's Progression Markers Initiative (PPMI) database (www.ppmi-info.org/data). sMRI data were acquired using 3.0 T Siemens scanners with 3D T1-weighted images. The acquisition parameters were as follows: TR = 2300 ms, TE = 3.0 ms, TI = 900 ms, flip angle = 9°, matrix size = 240 × 256, 176 sagittal slices, and 1 mm isotropic voxel. DMRI data were acquired using a single-shot echo-planar imaging (EPI) sequence with TR = 950 ms, TE = 88 ms, flip angle = 90°, 65 axial slices, 2 mm isotropic voxel, and field of view = 232 × 232 mm^2^. Diffusion weighting was applied along 64 non-collinear directions with a b-value of 1000 s/mm^2^, along with one b0 image. All diffusion images were acquired with a phase-encoding direction of posterior-to-anterior.

#### 2.1.2 Clinical assessments

All the participants underwent comprehensive clinical and neuropsychological assessments. Motor symptoms were assessed using the Movement Disorder Society-Unified Parkinson's Disease Rating Scale (MDS-UPDRS) parts I-III. The scores for bradykinesia, rigidity, and tremor symptoms were derived from UPDRS III as follows: the score for tremor was obtained from items 3.15, 3.16, item 3.17a-3.17c, and 3.18. The rigidity score was obtained from items 3.3a-3.3e. The bradykinesia score was obtained from items 3.4–3.9,3.14. Cognitive function was assessed using the Symbol Digit Modalities Test (SDMT), Delayed Verbal Test (DVT)-total recall score from Hopkins Verbal Learning Test (HVLT), and the Montreal Cognitive Assessment (MoCA). Depression was assessed using the Geriatric Depression Scale (GDS). Autonomic function was assessed using Scales for Outcomes in Parkinson's Disease-Autonomic Dysfunction (SCOPA-AUT) and was obtained from items 1–21. REM Sleep Behavior Disorder Questionnaire (RBDQ) was assessed using the REM-RBDQ. The age at onset, Hoehn and Yahr stage, and disease duration were recorded

### 2.2 Structural MRI pre-processing

T1-weighted images were processed using the free software Computational Anatomy Toolbox (CAT12) (http://www.neuro.uni-jena.de/cat/), within SPM12 (http://www.fil.ion.ucl.ac.uk/spm/software/spm12). CAT12 was chosen because it provides more advanced and computationally efficient brain segmentation with more accurate volumetric analysis than other available tools(Fu et al., 2020).

The processing pipeline included numerous automated steps: (1) skull stripping; (2) bias field correction; (3) tissue segmentation into gray matter (GM), white matter (WM), and cerebrospinal fluid (CSF) probability maps; and (4) spatial normalization to the Montreal Neurological Institute (MNI) standard space. The Harvard-Oxford cortical and subcortical atlases enabled regional volumetric analysis to extract data from 48 cortical and 21 subcortical regions.

### 2.3 Diffusion MRI preprocessing

Diffusion-weighted images (DWI) were denoised using MRtrix 3.0 (Tournier et al., 2019), followed by eddy current correction and brain extraction using FSL (FMRIB Software Library v6.0, http://www.fmrib.ox.ac.uk/fsl). Diffusion tensor fitting was performed using the FSL tensor fitting routine (DTIFIT) to generate fractional anisotropy (FA) and mean diffusivity (MD) maps. Each participant's FA map was non-linearly registered to the FMRIB58_FA_1mm template using a two-step procedure: affine registration via FLIRT, followed by non-linear warping with FNIRT.

The JHU-ICBM-labels-1 mm atlas (50 regions) and JHU-ICBM-tracts-prob-1 mm atlas (20 tracts) were inversely warped into the native diffusion space. For the tract-based analysis, probabilistic tract maps were thresholded at 25% and binarized to create individual tract masks; this threshold corresponds to retaining voxels in which the tract was identified in at least 7 of the 28 reference subjects used to construct the atlas, and matches the standard threshold distributed by FSL (JHU-ICBM-tracts-maxprob-thr25-1 mm) used to balance specificity against partial-volume contamination at tract margins. Mean values of FA and MD were extracted from all 70 white matter regions (50 ROIs from the labels atlas and 20 tracts from the tracts atlas).

### 2.4 Clustering analysis

The clustering pipeline consisted of five steps: feature definition, normative confounding adjustment, feature selection, dimensionality reduction, and hierarchical clustering.

#### 2.4.1 Feature definition

The full feature set comprised 209 imaging features: cortical volumes of 48 regions, subcortical volumes of 21 regions, mean FA values across 70 white matter regions, and mean MD across the same 70 regions.

#### 2.4.2 Normative interaction-aware confound adjustment

To isolate disease-specific neuroanatomical variations from variance attributable to demographic factors, total brain size, and scanner-related measurement effects, each imaging feature was adjusted using a normative modeling approach defined by the 40 NC participants. For each feature, a linear regression was fitted on the combined PD and NC sample with the following covariates:

$$f=\beta ₀+\beta ₁\cdot \mathrm{age}+\beta ₂\cdot \mathrm{sex}+\beta ₃\cdot \text{education}+\beta ₄\cdot \mathrm{TIV}+\beta ₅\cdot \text{scanner}+\beta ₆\cdot \text{group}+\beta ₇\cdot \left(\mathrm{age}\times \text{group}\right)+\varepsilon .$$

where f is is the imaging feature value being modeled (with the adjusted feature subsequently defined as the residual from this model under the normal control reference), TIV is total intracranial volume, group = 1 for PD, 0 for NCs, age × group is the age-by-group interaction term, scanner is a binary covariate coding scanner model (TrioTim vs non-TrioTim), and ε is the error term. TIV was excluded from the model for DTI features.

Adjusted feature values for each PD participant were computed as residuals from the predicted value under the normal control reference (group = 0 for NC, age × group = 0). The age × group interaction term was included to capture potential disease-specific modifications of normal aging effects, a critical consideration in neurodegenerative conditions where pathological processes may accelerate or alter typical aging trajectories.

#### 2.4.3 Feature selection

The adjusted features were normalized using RobustScaler, which centers each feature at its median and scales it by its interquartile range (IQR). Under this scaling, an approximately normally distributed feature has a post-scaled variance of approximately 0.55 (since IQR ≈ 1.35σ for normal data). Features with variance below a threshold of 0.5 were removed; this filter targets features dominated by extreme outliers (which RobustScaler compresses) or with severely limited dynamic range, while retaining features whose variability is consistent with that of a typical normally distributed feature. A total of 177 features were retained for subsequent analysis.

#### 2.4.4 Dimensionality reduction and clustering

**Fig. 1:**
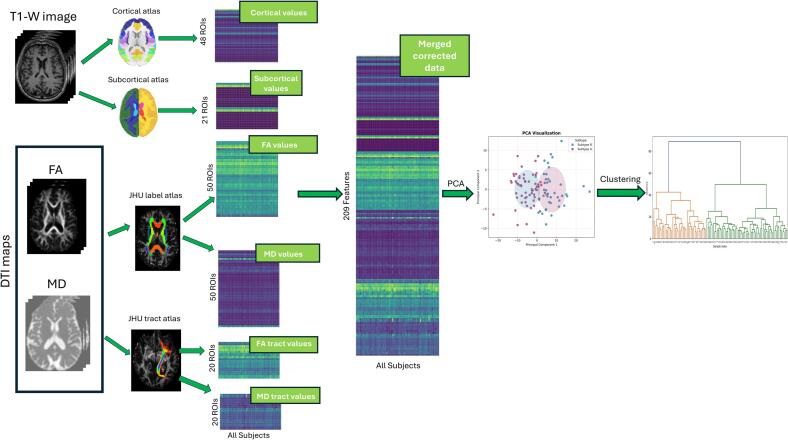
Pipeline used to extract features required in the clustering process. MD: mean diffusivity, and FA: fractional anisotropy.

The selected features were standardized and reduced using principal component analysis (PCA), retaining components contributing more than 1% of the total variance (18 components, cumulative variance 78.2%). Hierarchical clustering using Ward's linkage and Euclidean distance was performed on the principal component scores. Our overall pipeline is illustrated in Fig. 1. The optimal number of clusters was determined using the Silhouette and Calinski–Harabasz indices. Cluster reproducibility was evaluated using k-means clustering, yielding a 93% agreement between the cluster assignments from the two algorithms and bootstrap resampling.

#### 2.4.5 Robustness and sensitivity analyses

Four robustness analyses were performed to verify that the cluster solution did not depend on specific methodological choices. First, the variance threshold was swept across 0.3, 0.4, 0.5, and 0.6, retaining 209, 207, 177, and 114 features respectively; the two-cluster solution was preserved with ARI ≥ 0.71 against the reported solution at all four thresholds. Second, the number of retained principal components was swept from 5 to 30; the cluster structure was stable in the immediate neighbourhood of the selected count (18 components), with ARI ≥ 0.74 at four of six counts in the range 15–20. Third, two confound-ablation analyses tested whether the cluster structure was driven by the normative adjustment: removing the age × group interaction term yielded ARI = 0.32 against the main solution, and clustering on raw (non-residualised) features yielded ARI = 0.08, demonstrating that the cluster structure emerges from the normative residualisation rather than from the raw imaging features. Fourth, to test whether the cluster solution reflected a cross-sectional cognitive severity gradient rather than imaging-defined structure independent of cognitive performance, the SDMT score was added as an additional covariate in the normative regression and the entire pipeline was re-run; the resulting clustering preserved cluster sizes (*n* = 59, *n* = 44) and showed ARI = 0.85 against the main solution, indicating that the clusters are not a cognitive-performance readout. Full sensitivity-analysis results, including the confusion matrix for the SDMT-decoupled clustering and PCA-space visualisations of the confound ablations, are reported in Supplementary Figs. S4-S5.

### 2.5 Statistical analysis

Demographic and clinical differences between identified subtypes were assessed using independent *t*-tests for continuous measures and chi-square tests for categorical variables (sex, Hoehn & Yahr stage (H&Y)), followed by general linear models (GLM) adjusting for age, sex, and education years. H&Y stage was additionally dichotomized and analyzed using logistic regression. Multiple comparisons were corrected using the Benjamini–Hochberg false discovery rate (FDR) procedure.

Two sets of analyses were conducted to delineate the biological characteristics of the identified PD subgroups. First, for comparisons between the two subtypes, Cohen's d effect sizes were computed for each imaging feature. Inferential testing of the same features used to construct the clusters is statistically circular (post-selection inference), and *p*-values are therefore not reported for between-subtype contrasts. Second, for comparisons of each subtype against NCs, formal hypothesis testing was performed using general linear models with appropriate covariate adjustments. For structural features (cortical and subcortical volumes), the model included age, sex, education years, TIV, and scanner model. For DTI features (FA and MD), the model included age, sex, education years, and scanner model (TIV was not included for DTI). All models were fitted separately for each imaging feature, and p-values for group differences were corrected for multiple comparisons within each modality using FDR correction. This approach provides robust statistical evidence for subtype abnormalities relative to healthy aging while appropriately characterizing subtype differences using effect size metrics.

To investigate the clinical relevance of the imaging signatures, partial correlation analyses were performed between selected imaging features and clinical outcome measures (SDMT and age at onset). Given the high dimensionality of the feature set, a feature-selection step was applied prior to correlation testing to prioritize the most discriminative biomarkers and reduce the multiple comparison burden. For features distinguishing between the two PD subtypes, features with Cohen's d > 0.8 (conventionally interpreted as a large effect size) were retained. For features distinguishing each subtype from NC, features with FDR-corrected *p* < 0.05 were retained. To further constrain dimensionality, the top 10 features per modality (cortical volume, subcortical volume, regional FA, regional MD, tract FA, and tract MD) were retained for each comparison; where fewer than 10 features met the threshold, all qualifying features were retained. Partial correlations between the selected imaging biomarkers and clinical scores were computed using pingouin.partial_corr (Python), controlling for age, sex, education years, and scanner model, with the addition of TIV for structural features. *P*-values were corrected using the FDR correction within each clinical variable.

### 2.6 Longitudinal cognitive trajectory analysis

The longitudinal cognitive trajectory was assessed using a linear mixed-effects (LME) model with the MoCA total score as the dependent variable. The model included fixed effects for subtype, time (in years from baseline), their interaction, and covariates (age at baseline, sex, and education years), with a random intercept per participant. The subtype × time interaction tested whether the rate of cognitive change differed between subtypes; this interaction term is the primary inferential test, with the within-subtype slopes (β₁ for Subtype A, β₂ for Subtype B) reported as descriptive trajectory estimates. Missing intermediate visits were handled natively by maximum likelihood estimation under a missing-at-random assumption; participants with fewer than two longitudinal observations were excluded as the slope could not be estimated from a single time point (*n* = 3 participants excluded). The models were fitted using the maximum likelihood estimation in statsmodels (Python). In a sensitivity analysis, the age at onset was substituted for age at baseline to test whether any trajectory difference reflected disease-timing biology rather than independent subtype-related decline.

## 3 Results

Hierarchical clustering identified a two-cluster solution as optimal based on the silhouette (0.22) and Calinski–Harabasz (38.4) indices (Supplementary Fig. S1). Validation using k-means clustering demonstrated high stability, with 93% agreement between methods (Supplementary Fig. S8). Subtype A included 59 participants and Subtype B included 44 participants.

### 3.1 Demographic and clinical characteristics

**Fig. 2:**
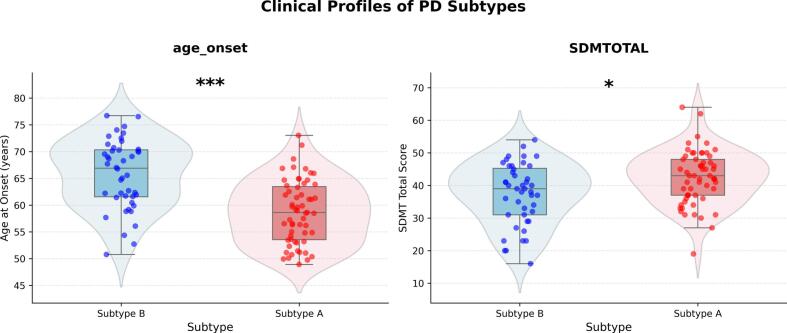
Differences in clinical features between the two PD subtypes at baseline.

A significant difference in age was observed between subtypes (*p* < 0.001), with participants in Subtype B being approximately 7 years older than those in Subtype A. Sex and education years did not differ between subtypes. Age at onset also differed significantly: participants with Subtype A had an earlier disease onset (58.7 ± 5.95 years) than those with Subtype B (65.73 ± 6.45 years; p < 0.001). Subtype A exhibited a milder cognitive profile, with significantly higher SDMT performance (adjusted *p* = 0.02; Fig. 2), indicating a better cognitive processing speed. No significant between-subtype differences were observed for tremor, bradykinesia, rigidity, UPDRS Parts II and III total scores, or non-motor assessments. The full demographic and clinical characteristics are presented in Table 1.

**Table 1:** Demographics and clinical characteristics of Parkinson's disease patients in the two subtypes.

Clinical Variable	Normal Control(*n* = 40)	Subtype A(n = 59)	Subtype B(n = 44)	*P* value	Adj. P
Demographics					
Age, years	63.46 ± 7.24	60.73 ± 6.38	67.73 ± 6.08	**<0.001**	/
Sex, (male/female)	22/18	36/23	33/11	0.2	/
Education, years	15.25 ± 2.54	15.07 ± 2.82	15.34 ± 3.06	0.644	/
HY stage (HY 1/HY 2)	/	28/31	13/31	0.1	0.52
Age onset	/	58.7 ± 5.95	65.73 ± 6.45	**<0.001**	**<0.001**
Disease duration	/	1.83 ± 1.37	2.41 ± 3.14	0.2	0.52
Motor					
Rigidity Score	0.1 ± 0.63	3.9 ± 2.49	5.07 ± 2.98	**0.03**	0.17
Bradykinesia Score	0.18 ± 0.50	10.42 ± 5.4	11.39 ± 6.6	0.42	0.66
Tremor Score	0.125 ± 0.46	3.61 ± 2.97	4.0 ± 3.52	0.54	0.66
UPDRS II	0.18 ± 0.64	5.37 ± 3.7	5.59 ± 3.61	0.77	0.78
UPDRS III	0.4 ± 1.34	21.32 ± 8.61	22.57 ± 11.39	0.53	0.66
Non-motor					
UPDRS I	2.08 ± 3.43	4.93 ± 3.44	5.41 ± 4.05	0.52	0.66
SDMT (total)	46.1 ± 9.96	42.41 ± 8.3	37.41 ± 9.46	**0.01**	**0.02**
SCOPA-AUT	6.15 ± 3.83	8.53 ± 8.07	10.25 ± 6.28	0.24	0.52
HVLT (DVT total recall)	48.45 ± 10.36	43.71 ± 11.13	46.8 ± 11.13	0.19	0.52
MoCA (total)	28.23 ± 1.07	27.27 ± 2.16	27.39 ± 2.07	0.79	0.78
REM-RBDQ	3.25 ± 2.10	3.78 ± 2.48	4.18 ± 2.45	0.42	0.66
GDS	5.45 ± 1.34	5.22 ± 1.45	5.36 ± 1.56	0.63	0.73

For the comparison of Sex and NHY, the chi-square test was used. Other measurements were analyzed using ANOVA. All *P* values are for subtype A vs. subtype B. Adj: adjusted.

### 3.2 Biological characterization

Between-subtype comparisons revealed widespread structural and microstructural differences. Subtype B exhibited significantly reduced volumes compared to Subtype A angular gyrus and frontal and central opercular cortices, along with enlargement of the left lateral ventricle (Cohen's d > 0.8). Moderate effect sizes (Cohen's d > 0.5) were observed across additional regions including the amygdala, thalamus, and various temporal, frontal, and supramarginal gyri.

Direct subtype comparisons demonstrated significantly lower FA and higher MD in Subtype B across multiple white matter tracts. FA reductions in Subtype B were most pronounced in the corpus callosum (genu, body, and splenium), superior and inferior fronto-occipital fasciculi, bilateral anterior thalamic radiation, forceps minor and major, right sagittal stratum, cingulum, right inferior longitudinal fasciculus, internal capsule segments, cerebellar peduncles, and right tapetum. MD increases were detected in largely overlapping tracts, with additional involvement of the fornix (including crus), corticospinal tract, and external capsule, without corresponding FA reductions.

Compared with NC, Subtype A exhibited no significant volumetric differences. However, it demonstrated widespread increases in FA (FDR p < 0.001 to 0.021) across 6 commissural fibers (including corpus callosum subdivisions, forceps major and minor, and right tapetum), 15 association pathways (including bilateral superior and inferior fronto-occipital fasciculi, superior and inferior longitudinal fasciculi, uncinate fasciculus, sagittal stratum, and cingulum), 17 projection fibers (anterior and posterior thalamic radiations, corona radiata, corticospinal tract, and internal capsule segments), and 6 brainstem pathways (middle, superior, and inferior cerebellar peduncles, and cerebral peduncles). Corresponding decreases in MD (FDR p ≤ 0.023) were observed in many overlapping tracts, including the bilateral superior longitudinal fasciculus (main body and temporal part), bilateral inferior fronto-occipital fasciculus, and cingulum.

**Fig. 3:**
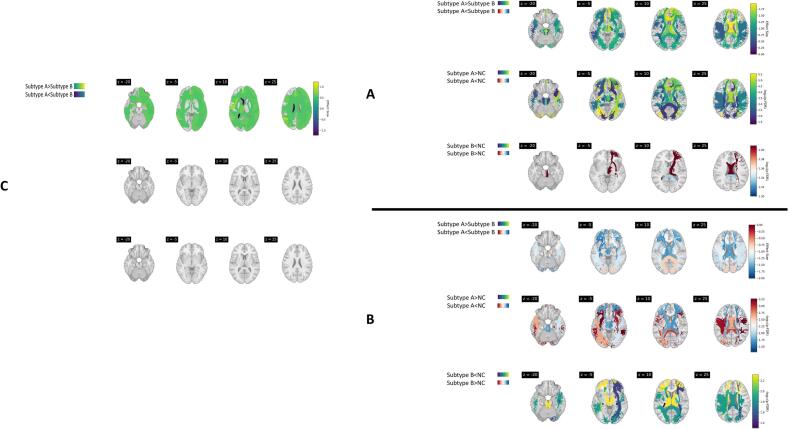
Differences in brain regions among the groups. To compare subtypes versus normal controls, biological features were adjusted for age, sex, education, and scanner model, and additionally, TIV for sMRI features, and all *p*-values were corrected using FDR correction. Effect size was used within the subtype comparison to compare subtypes with each other. a: for fractional anisotropy comparison; features, b: for mean diffusivity comparison, and c: for sMRI features.

Subtype B versus NC demonstrated no significant volumetric reductions. However, several regions showed trend-level reductions that did not survive correction (FDR p ≈ 0.065), including the central and frontal opercular cortices, inferior frontal gyrus (pars opercularis and triangularis), parahippocampal gyrus, inferior temporal gyrus (posterior division), middle temporal gyrus (temporooccipital part), along with enlargement in the left lateral ventricle. Significant reductions in FA were less widespread than MD changes, including the corpus callosum (genu and body), right tapetum, left superior fronto-occipital fasciculus, and right anterior thalamic radiation (FDR p = 0.039 to 0.046). Widespread significant increases in MD were observed, involving 11 association fibers, 15 projection fibers, 5 commissural fibers, and the middle cerebellar peduncle (all FDR p  = 0.004 to 0.049). As shown in Fig. 3.

### 3.3 Correlations between biological and clinical parameters

Partial correlation analyses were performed between 42 selected imaging biomarkers and the two clinical variables (SDMT and age at onset), controlling for age, sex, education, scanner model, and total intracranial volume (for structural features). Six features showed significant FDR-corrected correlations with age at onset in the full PD sample: bilateral amygdala volumes, middle temporal cortex (temporooccipital part) volume, and microstructure (MD) of right superior corona radiata, right superior longitudinal fasciculus, and right superior longitudinal fasciculus temporal part. Stratified by subtype, no correlations survived FDR correction within either group. Within Subtype A, five features showed moderate-magnitude correlations that did not reach FDR significance (*r* = 0.30 to 0.42, raw *p* < 0.01, FDR ≈ 0.06); the same features showed weaker or null correlations within Subtype B (all *r* < 0.26, raw *p* > 0.11). No correlations between SDMT and imaging features survived FDR correction at any level. The full correlation results are provided in Supplementary Fig. S6.

### 3.4 Validation of PD clusters

Cluster reproducibility was evaluated using bootstrap resampling (100 iterations). For each iteration, the participant pool was resampled with replacement, and the full clustering pipeline was rerun on the resampled data. The feature profile of each sample was then assessed, and finally, the z-score of each feature was obtained. Finally, the z-scores of all feature profiles were averaged and compared with our original feature profile. Feature-wise z-score profiles showed strong correlations between the original and validation datasets (*r* = 0.99 for cluster 0; r = 0.99 for cluster 1; Supplementary Fig. S7). Therefore, the biological signatures distinguishing the subtypes were highly reproducible under participant resampling.

### 3.5 Longitudinal clinical relevance

**Fig. 4:**
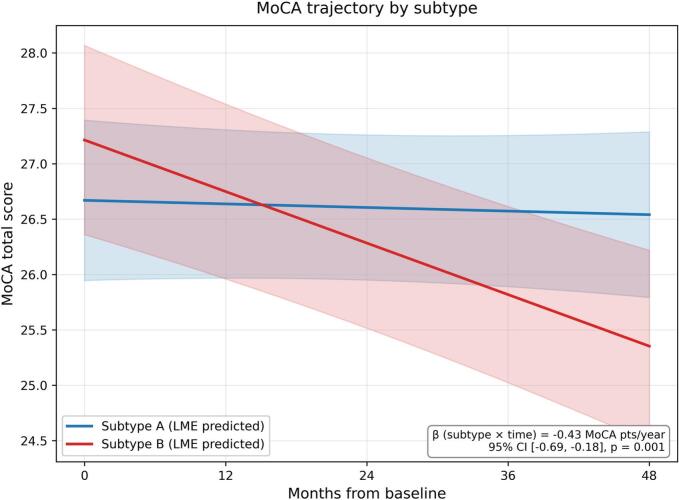
Cognitive trajectory across the 48-month follow-up for the two PD subtypes. Predicted trajectories from the linear mixed-effects model are shown as solid lines with 95% confidence bands.

The two subtypes exhibited significantly different cognitive trajectories during the 48-month follow-up. The linear mixed-effects model revealed that the average rate of MoCA change within Subtype A was minimal (β₁ = −0.03 points/year, *p* = 0.70), whereas Subtype B showed significant cognitive decline (β₂ = −0.47 points/year, *p* < 0.001). The subtype × time interaction (the primary inferential test of differential trajectory) was significant (β₃ = −0.43 points/year, 95% CI: −0.69 to −0.18, p < 0.001), indicating that Subtype B declined cognitively faster than Subtype A by approximately 0.4 MoCA points per year (Fig. 4). Over the full 48-month follow-up period, this translates to an estimated 1.7-point greater decline in Subtype B than in Subtype A. The trajectory difference was robust to the substitution of age of onset for age at baseline in the model (β₃ = −0.43, p < 0.001), demonstrating that the differential decline was not mediated by the age-of-onset differences between subtypes and reflected a genuine difference in the rate of cognitive change between the imaging-defined subgroups.

## 4 Discussion

In this study, we identified two imaging-defined Parkinson's disease subtypes using an unsupervised multimodal neuroimaging framework that integrated diffusion MRI and structural MRI features within a normative, interaction-aware confound-adjustment framework. The subtypes differed not merely along a single severity axis but along qualitatively distinct patterns of brain structural organization. Subtype A exhibited widespread FA increases, MD reductions, preserved cortical and subcortical volumes, and a milder cross-sectional cognitive profile. Subtype B showed diffuse MD elevations, limited FA reductions, trend-level cortical volume reductions, ventricular enlargement, and lower cognitive performance. Together, these patterns suggest that early PD heterogeneity is not adequately captured by a single severity axis but instead encompass qualitatively distinct combinations of macrostructural preservation, microstructural alteration, and clinical course. Robust cluster stability was demonstrated under both cross-algorithm validation (93% agreement between hierarchical clustering and k-means) and bootstrap resampling of cluster centroids (r ≈ 0.99 for both subtypes), supporting the robustness of the two-cluster solution.

The microstructural signature of Subtype A, widespread FA elevations and corresponding MD reductions across commissural, association, projection, and brainstem pathways superficially resembles a pattern of microstructural preservation. Prior studies have reported increased anisotropy and fiber density in motor-related tracts during early disease stages(Mole et al., 2016; Shih et al., 2023). Our findings extend this literature by demonstrating pronounced FA increases and MD decreases not only in motor pathways (e.g., corticospinal tract, internal capsule, cerebellar peduncles) but also widening to encompass networks supporting attention, executive function, processing speed, memory, including superior and inferior fronto-occipital fasciculi, superior longitudinal fasciculus, anterior thalamic radiation, and anterior corona radiata. This pattern aligns with the cross-sectional preservation of cognitive processing speed in Subtype A (higher SDMT relative to Subtype B) and with the stable MoCA trajectory observed over the 48-month follow-up. The absence of significant volumetric atrophy in this subtype aligns with previous work (Gattellaro et al., 2009), which established that microstructural white matter changes often precede macrostructural alterations, making DTI a more sensitive biomarker for early-stage stratification.

The microstructural profile of subtype A, featuring widespread FA increases and MD decreases, may reflect genuine compensatory or adaptive microstructural reorganization in response to early dopaminergic depletion, potentially involving increased fiber density or myelination in specific pathways, which supports the preserved cognitive trajectory observed clinically. However, alternative biological mechanisms must be considered. Prior work has shown that, the majority of cerebral white matter voxels contain crossing fibers, and FA is highly sensitive to the relative volume fractions of these fiber populations as well as their individual microstructural integrity. Disproportionate degradation of one fiber bundle alongside relative preservation of another can produce paradoxical FA increases without any actual increase in microstructural integrity. Therefore, the elevated FA can be interpreted as a selective loss of crossing fibers with relative preservation of dominant pathways(Figley et al., 2022). Without complementary measurements robust to crossing-fiber configurations, such as neurite orientation dispersion and density imaging (NODDI) or fixel-based analysis, we cannot adjudicate among these possibilities. We therefore characterize Subtype A's microstructural signature as FA-preserved.

Subtype B exhibited a pattern more consistent with diffuse white matter degeneration in advancing PD pathology(Owens-Walton et al., 2024; Yang et al., 2023) with anatomically specific implications for cognitive function. Significant FA reductions in Subtype B affected a set of tracts supporting inter-hemispheric integration, executive function, and semantic processing, including corpus callosum, bilateral superior and inferior fronto-occipital fasciculi, and bilateral anterior thalamic radiations. MD elevations were even more widespread, involving largely overlapping tracts with additional involvement of the fornix (including the fornix crus) and external capsule. The fornix finding is particularly relevant for cognitive decline, as this tract is the principal hippocampal efferent pathway supporting episodic memory; alteration of this tract is consistent with progressive cognitive impairment, including the type of decline measured by MoCA, which incorporates memory subscales. A notable feature of the Subtype B signature was that widespread MD increases were not always accompanied by commensurate FA reductions, a pattern consistent with neuroinflammation or axonal rarefaction in which expansion of the extracellular compartment elevates diffusivity while overall fiber coherence is partially preserved. Trend-level volumetric reductions suggest emerging macrostructural consequences of sustained microstructural change. The combination of widespread MD alterations with limited and regionally specific FA reductions suggests that Subtype B is best understood as a diffuse microstructural alteration phenotype rather than a phenotype defined by frank tract-specific degeneration alone.

The age-of-onset correlations observed in the full PD sample fall into two anatomically coherent groups: limbic-temporal volumes (bilateral amygdala, middle temporal cortex) and right-hemisphere association-tract microstructure. These full-sample findings indicate that imaging-defined brain structure varies systematically with disease-onset timing in PD. The subtype-stratified analyses indicate that this age-of-onset signal is concentrated within Subtype A: within this subtype, the same anatomical regions showed moderate-magnitude effects (*r* = 0.30 to 0.42) that approached but did not reach within-subgroup FDR significance, whereas Subtype B showed no comparable association. The lack of formal within-subtype significance reflects the limited statistical power of FDR-corrected subgroup testing at *n* = 59 and *n* = 44 rather than a true null effect, as supported by the consistent direction. Within Subtype A, age at onset therefore appears to index biologically meaningful variation in limbic and association-tract organization, plausibly related to the same processes underlying its FA-preserved microstructural signature and its stable longitudinal cognitive trajectory. The relative absence of equivalent correlations in Subtype B suggests a partial decoupling between regional pathology and disease timing once microstructural alterations become diffuse, a pattern consistent with the proposition that imaging-defined PD subtypes follow differing trajectories of clinical–anatomical correspondence.

The imaging-defined subtypes diverged significantly in their longitudinal cognitive trajectories over the 48-month follow-up. Subtype A exhibited essentially stable Montreal Cognitive Assessment (MoCA) scores (β₁ = −0.03 points/year, *p* = 0.70), whereas Subtype B showed significant cognitive decline (β₂ = −0.47 points/year, *p* < 0.001). The differential rate of decline (β₃ = −0.43 points/year, 95% CI: −0.69 to −0.18, p < 0.001) was robust to substitution of age at onset for age at baseline as a covariate, indicating that the trajectory difference cannot be attributed solely to differences in age at onset between subtypes. Over the full follow-up window, this differential rate corresponded to an estimated 1.7-point greater decline in Subtype B than in Subtype A. This longitudinal finding provides direct evidence that the imaging-defined subtypes carry prognostic information about cognitive course beyond their cross-sectional differences. It also informs interpretation of the FA-preserved signature in Subtype A: this group showed not only better cross-sectional cognitive performance but also preserved cognitive trajectory over four years.

Our findings align with prior work identifying malignant and mild PD phenotypes based on clinical and cognitive data, while extending these frameworks through purely biological subtyping(Fereshtehnejad et al., 2017; Fereshtehnejad et al., 2015). Unlike clustering approaches that incorporate clinical variables directly (Cao et al., 2022), our approach minimizes circularity by relying exclusively on neuroimaging features for cluster definition. Few prior studies have integrated diffusion metrics into PD subtyping(Sejnoha Minsterova et al., 2020; Zhu et al., 2025). By jointly analyzing FA and MD alongside volumetric features, our work extends these findings by revealing subtype-specific diffusion signatures that are not captured by macrostructural measures alone.

Several methodological aspects of this study distinguish it from prior PD subtyping work. First, the normative interaction-aware confound-adjustment model includes an age-by-group interaction term that explicitly accounts for disease-modified aging effects. Unlike traditional confound regression that removes only chronological aging, this approach removes the average pathological aging trajectory of PD and isolates biologically meaningful deviations from that expected course, reframing subtyping as a deviation-based rather than severity-based classification. Second, we incorporated the scanner model as a categorical covariate in the normative regression to absorb scanner-related measurement variance, addressing a known source of confounding in multi-scanner PPMI work. Third, we demonstrated cluster reproducibility through cross-algorithm validation (93% agreement between hierarchical clustering and k-means) and bootstrap resampling of cluster centroid profiles (r ≈ 0.99 for both subtypes), establishing that the identified subtypes are robust biological phenotypes rather than algorithm-dependent artifacts.

The identification of imaging-defined PD subtypes has potential implications for prognosis and stratified clinical management. Subtype B participants, characterized by diffuse microstructural alterations and accelerated cognitive decline may represent a population at higher risk for early cognitive impairment who could benefit from intensified monitoring or earlier intervention with cognitive-domain–targeted therapies. Subtype A's signature of potential compensatory microstructural reorganization suggests these patients might benefit most from interventions targeting neuroplasticity, such as cognitive training or neuromodulation. Subtyping based on baseline multimodal imaging could also refine clinical-trial design by stratifying participants according to expected trajectory, improving the statistical power to detect subtype-specific therapeutic responses.

Several limitations should be acknowledged. The moderate sample size limits the statistical power of within-subgroup analyses, as reflected in the failure of age-of-onset correlations within-subtype A to survive FDR correction despite consistent directional effects with the full cohort correlations. Replication in larger cohorts is needed to establish the stability of the subtype-specific clinical–imaging relationships and to validate the clinical applications before they can be considered. Moreover, FA and MD are tensor-derived metrics that can be confounded by crossing-fiber architectures(Figley et al., 2022); complementary measurements with multi-fiber or microstructure-specific contrasts will be necessary to adjudicate among compensatory, selective-degeneration, and artifactual interpretations of the Subtype A FA-elevation pattern. Also, feature selection via variance thresholding and PCA may have omitted subtle signals, and clustering assumptions (Euclidean distance, Ward's linkage) may not capture non-linear relationships; graph-based or representation-learning approaches could be explored in future work. In addition, the small de novo, unmedicated cohort limits generalizability to advanced or medicated PD populations, and unmeasured biological factors such as genetic variants (e.g., GBA mutations) may influence subtype membership in ways our framework does not capture. Future work should incorporate complementary imaging modalities, including functional MRI, neuromelanin-sensitive MRI, and PET measures of dopaminergic integrity to enrich the biological characterization of the imaging-defined subtypes.

## 5 Conclusion

This study revealed two imaging-defined subtypes of de novo Parkinson's disease by leveraging a multimodal unsupervised clustering framework. The findings indicate that early PD heterogeneity is not adequately described by a single severity axis but instead reflects qualitatively distinct combinations of macrostructural and microstructural organization. Subtype A was characterized by widespread elevations in white matter fractional anisotropy, preserved cortical and subcortical volumes, milder cross-sectional cognitive impairment, and stable cognitive trajectory over the 48-month follow-up. Subtype B was characterized by diffuse increases in mean diffusivity, more limited and regionally specific FA reductions, trend-level volumetric reductions, ventricular enlargement, and significantly faster cognitive decline.

Methodologically, our results demonstrate the indispensability of multimodal feature fusion for biologically meaningful PD subtyping. Macrostructural measures alone are insufficient to capture early disease heterogeneity, as key pathological signatures are predominantly microstructural. The dissociation between fractional anisotropy and mean diffusivity further underscores the complementary value of diffusion metrics, with mean diffusivity emerging as a sensitive marker of diffuse tissue disruption in early PD. The normative interaction-aware confound-adjustment framework, the inclusion of scanner-related variance in the regression model, and the bootstrap demonstration of cluster reproducibility together support the validity of the identified subtypes. While elevated FA in Subtype A is compatible with a compensatory or pre-degenerative interpretation supported by its preserved cognitive trajectory, definitive biophysical interpretation will require complementary imaging contrasts. These findings provide a biologically grounded framework for understanding PD heterogeneity in the earliest disease stages and a foundation for future prognostic stratification.

## CRediT authorship contribution statement

**Esraa Arabi:** Writing – original draft, Validation, Methodology, Data curation, Conceptualization. **Yuan Tian:** Writing – review & editing. **Minwei Zhu:** Writing – review & editing, Funding acquisition, Formal analysis. **Wenpeng Gao:** Writing – review & editing, Supervision, Project administration, Investigation, Conceptualization. **Yili Fu:** Writing – review & editing, Funding acquisition.

## Declaration of competing interest

The authors declare that they have no known competing financial interests or personal relationships that could have appeared to influence the work reported in this paper.

## Data Availability

The authors do not have permission to share data.
