# Supplementary material for: Multimodal magnetic resonance imaging-based clustering identifies two imaging-defined brain phenotypes in De novo Parkinson's disease
Source: Neuroimage Clin. 2026 Jun 9;51:104021. doi: 10.1016/j.nicl.2026.104021 (PMC13277644; doi:10.1016/j.nicl.2026.104021)
Supplement: Supplementary file 1 — Supplementary material [file mmc1.docx]

**Supplementary Data**

Multimodal magnetic resonance imaging-based clustering identifies two imaging-defined brain phenotypes in De Novo Parkinson's disease

This supplementary data includes as follows:

**Supplementary Figures**

Figure S1. The quality of hierarchical clustering using the silhouette score and the Calinski-Harabasz score

Figure S2. Sensitivity of cluster solution to different variance threshold

Figure S3. Validation of PCA components choice

Figure S4. Severity decoupling: agreement between main and SDMT-residualised clustering.

Figure S5. Confound-adjustment ablation.

Figure S6. Correlations between clinical parameters and imaging features

Figure S7. Clusters profile stability.

Figure S8. Confusion matrix between hierarchical and k-means clustering

**Supplementary Tables**

Table S1. Demographics of normal controls and Parkinson’s disease patients in our dataset

Table S2. Significant features between subtype A and subtype B

Table S3. Significant features between subtype A and subtype NC

Table S4. Significant features between subtype B and subtype NC

Table S5. Features selected for correlation analysis

**Supplementary Figures**

**
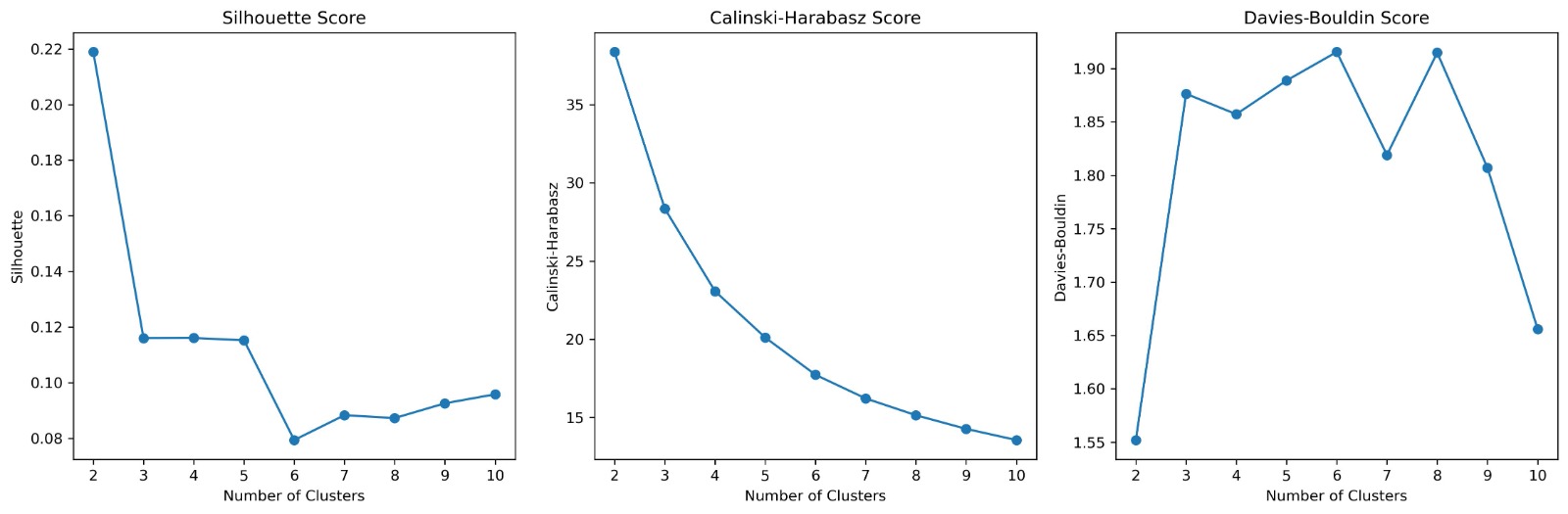
**

**FIGURE S1** | The quality of Hierarchical clustering using silhouette score and Calinski-Harabasz score


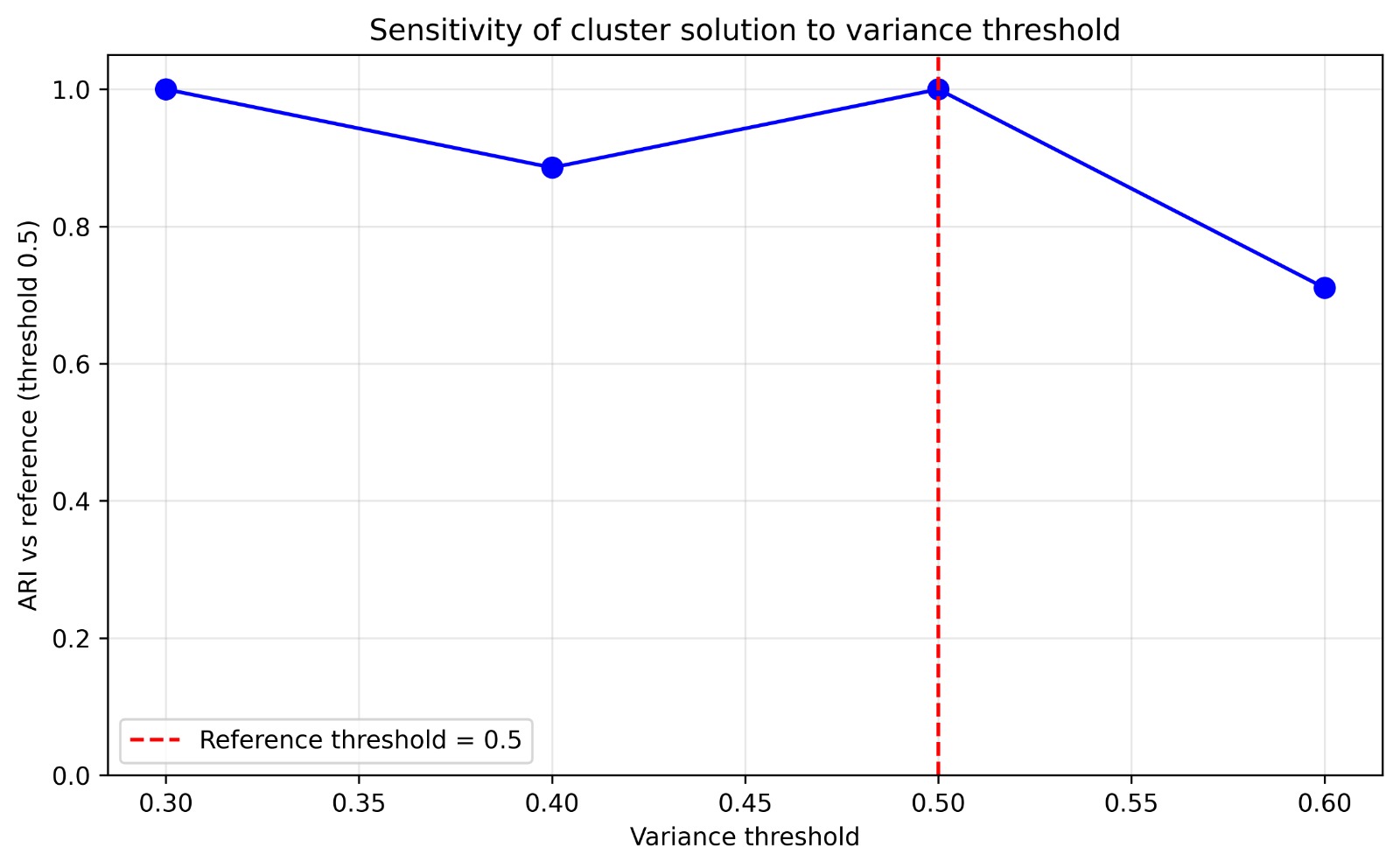


**FIGURE S2** | sensitivity of cluster solution to different variance threshold


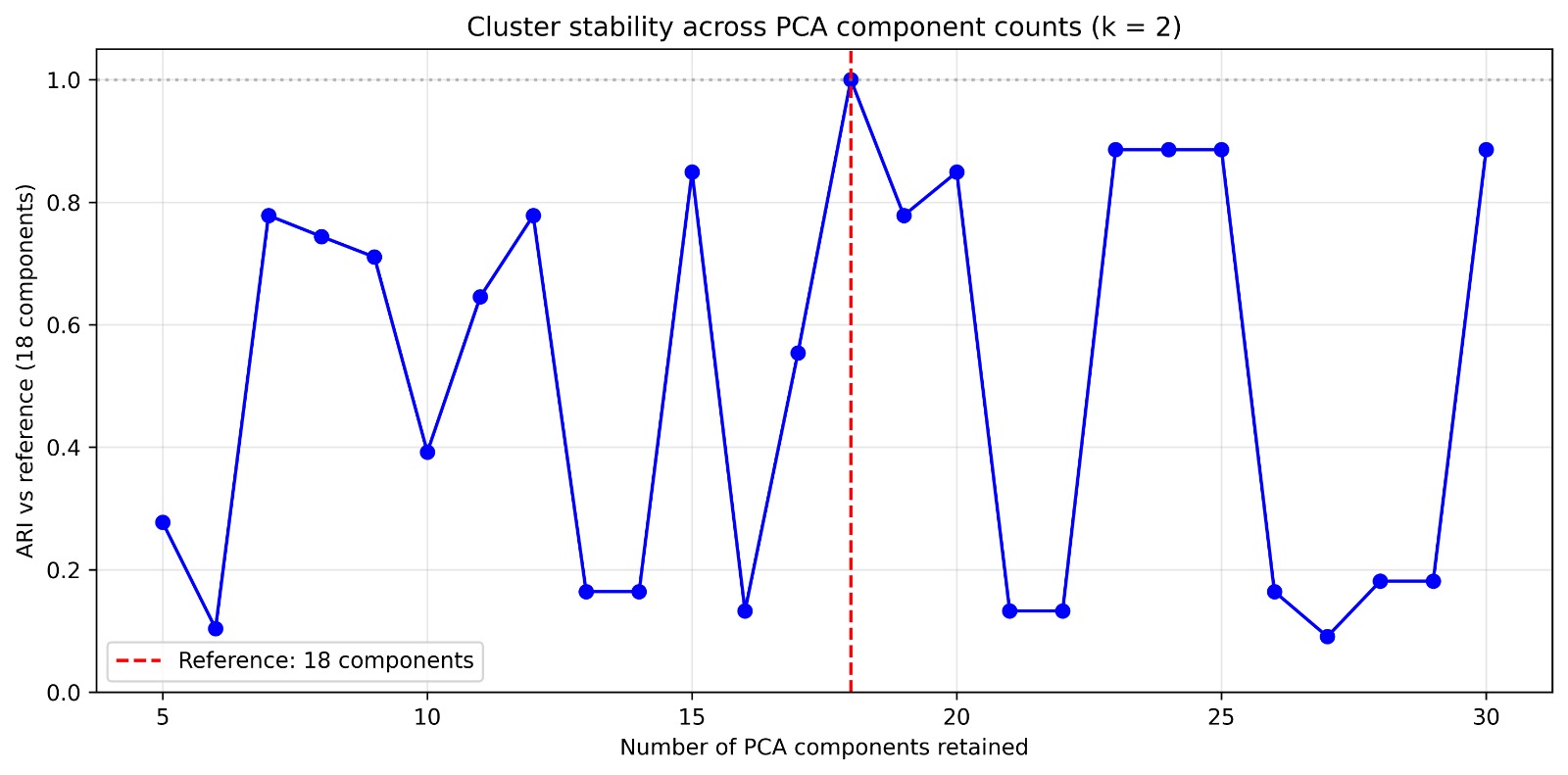


**FIGURE S3|** Validation of PCA components choice

**
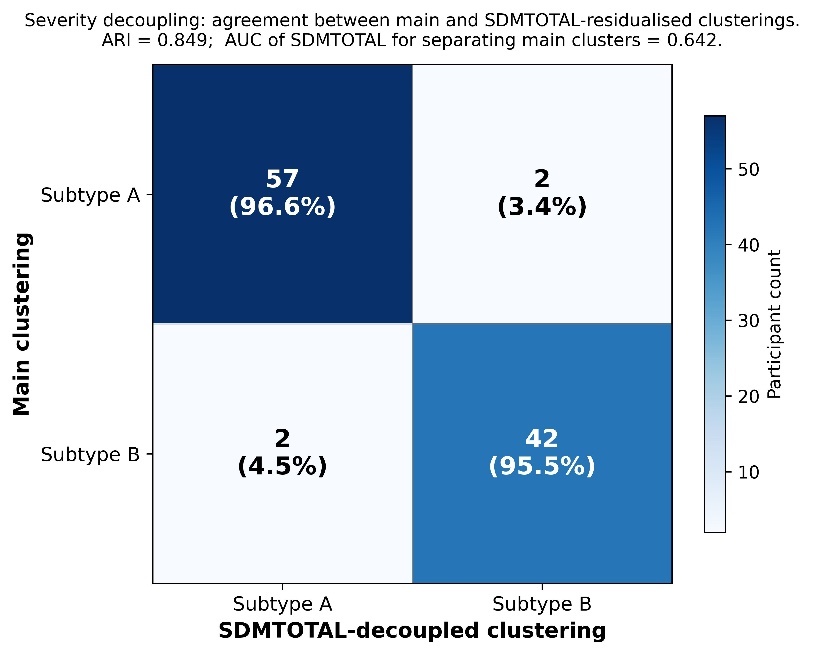
**

**FIGURE S4|** Severity decoupling: agreement between main and SDMT-residualised clustering.

**
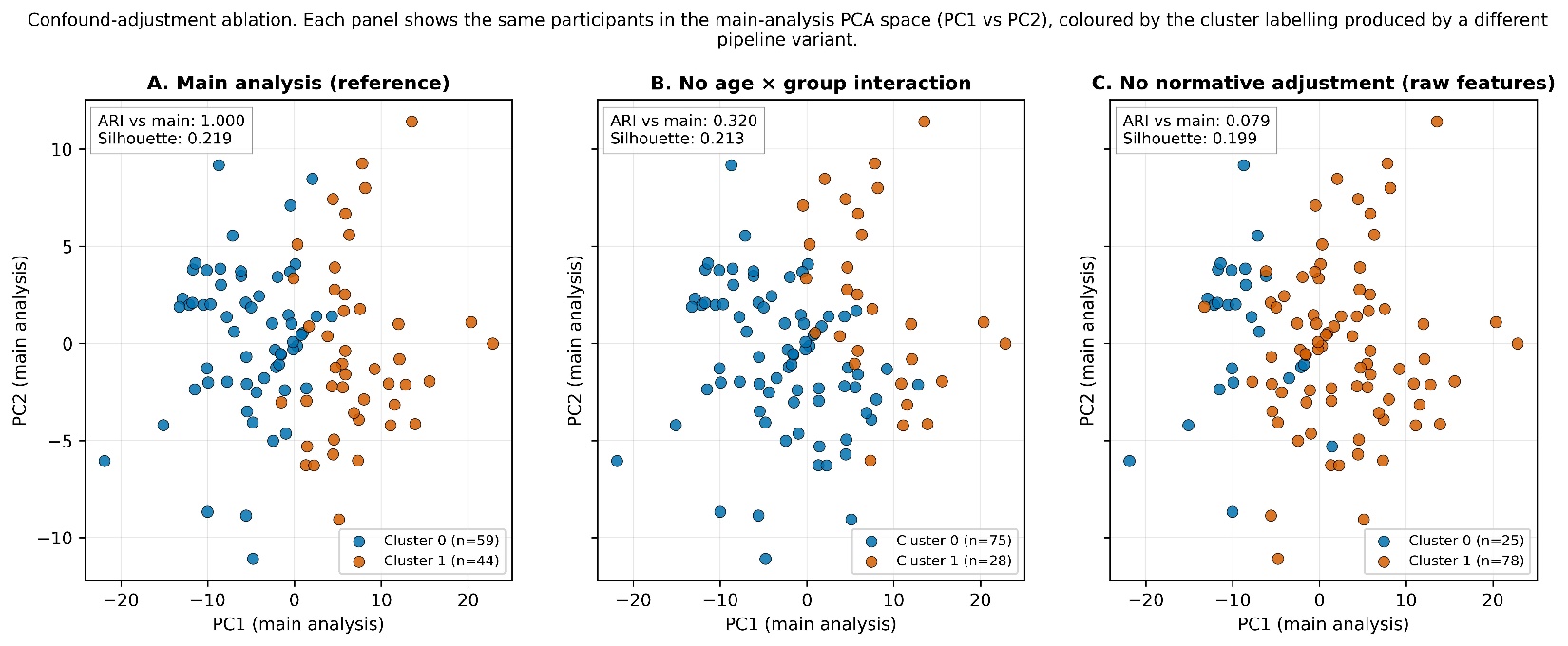
**

**FIGURE S5|** Confound-adjustment ablation. Each panel shows the same participants in the main-analysis PCA space (PC1 vs PC2), colored by the cluster labelling produced by a different pipeline variant.

| **A** | **B** |
| --- | --- |
| 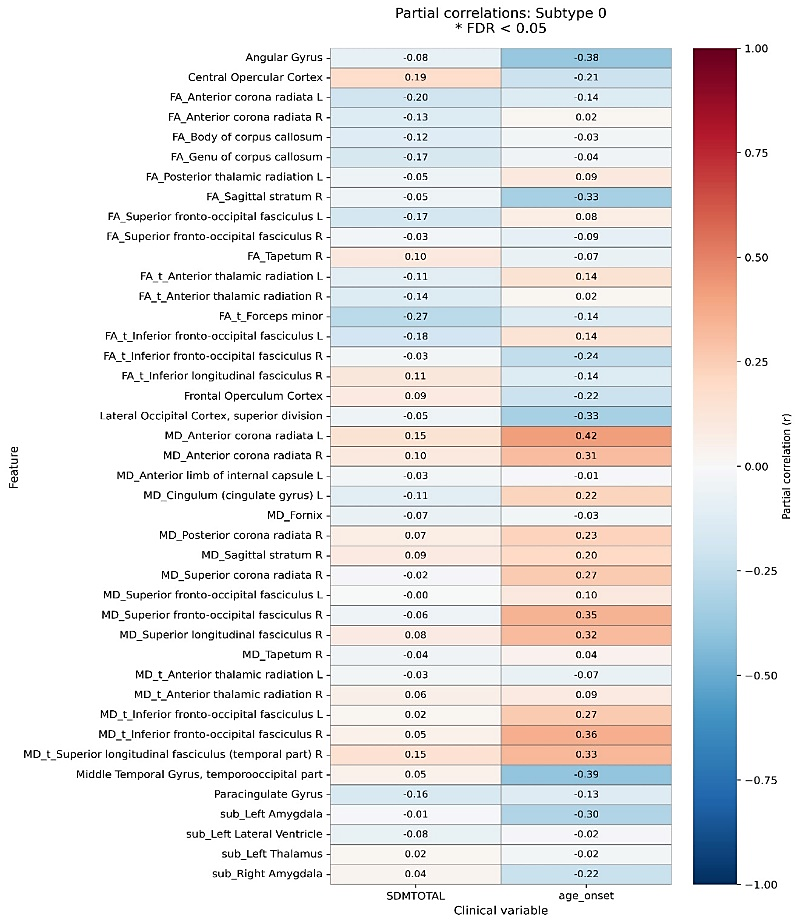 | 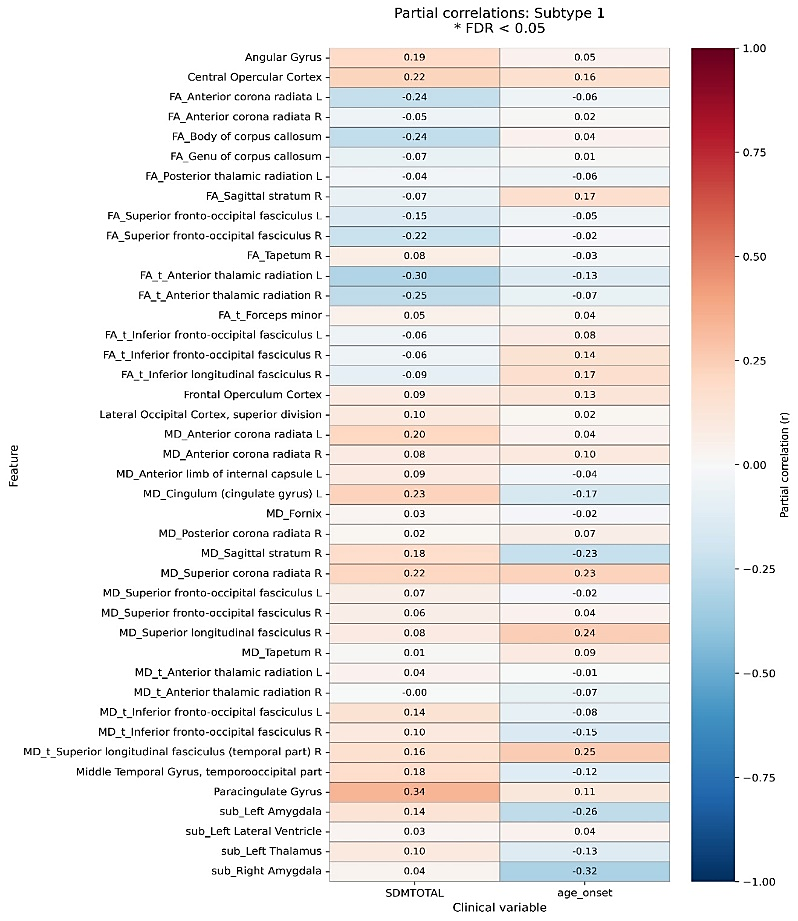 |
| C | |
| 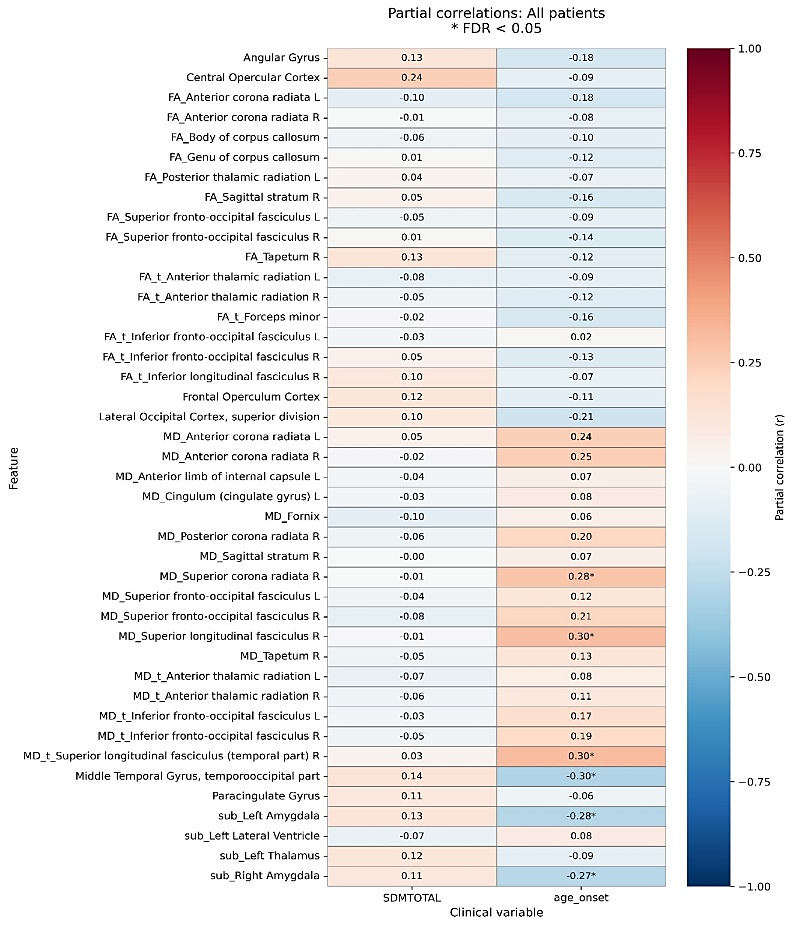 | |

**FIGURE S6 |** Correlations between clinical parameters and imaging features. A. Heatmap explaining the correlation between imaging attributes and the clinical measures for subtype A. B. Heatmap explaining the correlation between imaging attributes and clinical measures for subtype B. C. Heatmap explaining the correlation between imaging attributes and clinical measures for all PD patients. All the imaging features were corrected for age, sex, education and additionally TIV for sMRI features only, followed by FDR test for all p-values from the correlation analysis. * for FDR<0.05.

| 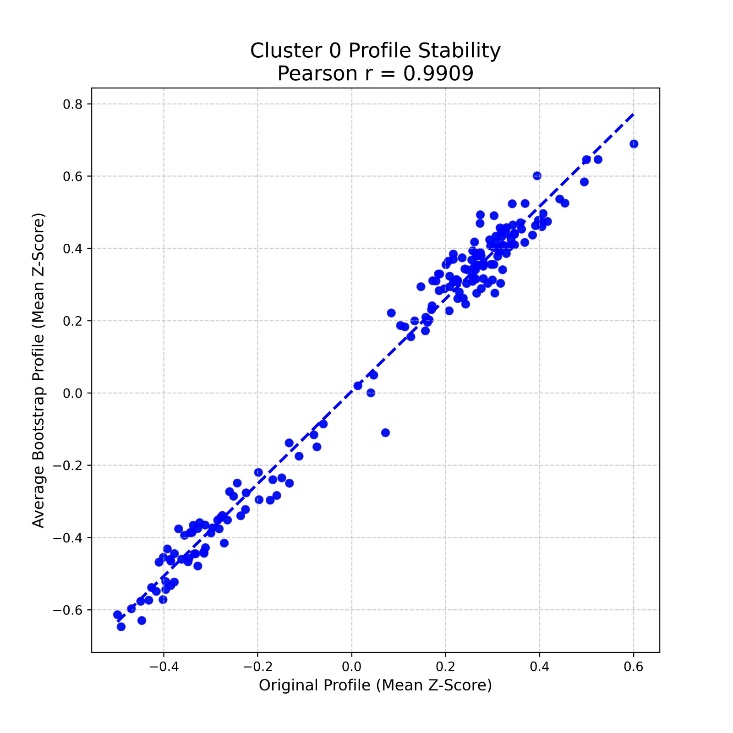 | 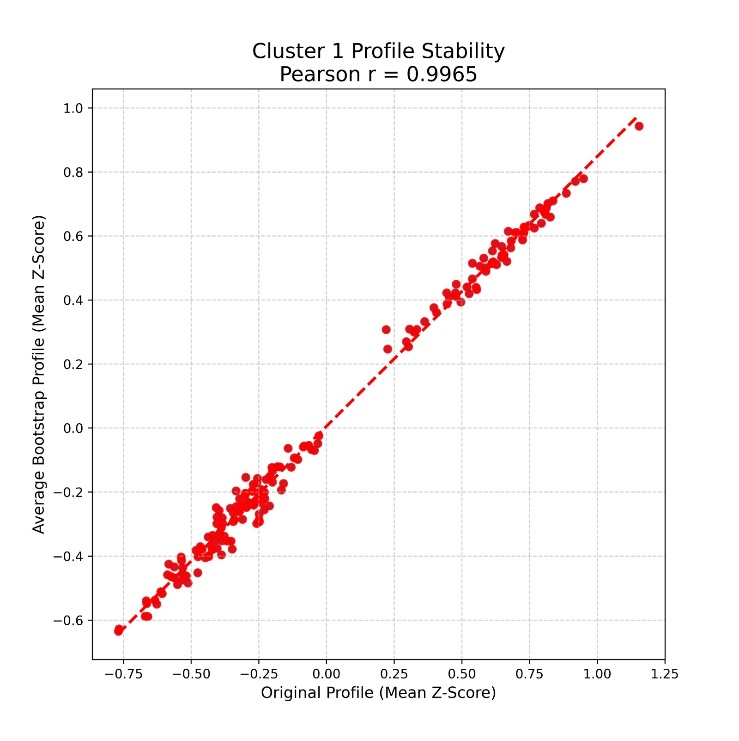 |
| --- | --- |

**FIGURE S7** | Clusters Profile stability.


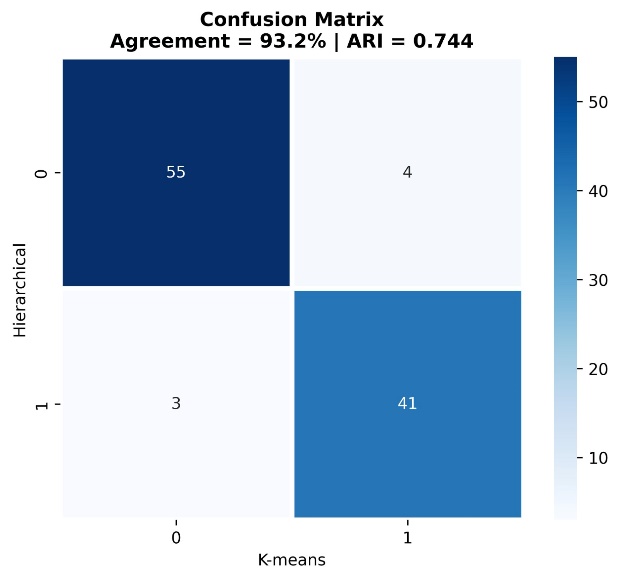


**FIGURE S8|** Confusion matrix between hierarchical and k-means clustering

**Supplementary Tables**

**TABLE 1** | Demographics of normal controls and Parkinson’s disease patients in our dataset

| Clinical Variable | Parkinson’s disease | Normal control |
| --- | --- | --- |
| **Demographics** |  |  |
| Subjects | 103 | 40 |
| Age, years | 63.72±7.13 | 63.46±7.24 |
| Sex, (male/female) | 69/34 | 22/18 |
| Education, years | 15.18±2.9 | 15.25±2.54 |

**TABLE 2** | Significant features between subtype A and subtype B

| **sMRI volumes** | **Cohen’s d** |
| --- | --- |
| sub_Left Lateral Ventricle | -1.22092 |
| Angular Gyrus | 0.942153 |
| Frontal Operculum Cortex | 0.919887 |
| Central Opercular Cortex | 0.903051 |
| Paracingulate Gyrus | 0.765863 |
| sub_Right Amygdala | 0.740907 |
| Middle Temporal Gyrus, temporooccipital part | 0.719846 |
| sub_Left Amygdala | 0.71535 |
| sub_Left Thalamus | 0.69903 |
| Lateral Occipital Cortex, superior division | 0.694362 |
| Middle Frontal Gyrus | 0.67501 |
| Supramarginal Gyrus, posterior division | 0.66025 |
| Inferior Frontal Gyrus, pars opercularis | 0.648526 |
| Parahippocampal Gyrus, posterior division | 0.643786 |
| Intracalcarine Cortex | 0.636532 |
| Subcallosal Cortex | 0.636143 |
| sub_Right Cerebral Cortex | 0.632559 |
| Superior Temporal Gyrus, posterior division | 0.632418 |
| Inferior Temporal Gyrus, temporooccipital part | 0.606691 |
| Inferior Frontal Gyrus, pars triangularis | 0.603775 |
| sub_Left Caudate | 0.593805 |
| Supramarginal Gyrus, anterior division | 0.585891 |
| Middle Temporal Gyrus, posterior division | 0.585184 |
| Planum Polare | 0.580031 |
| Cingulate Gyrus, anterior division | 0.579872 |
| Frontal Orbital Cortex | 0.576472 |
| Postcentral Gyrus | 0.566066 |
| sub_Left Cerebral White Matter | 0.562911 |
| sub_Right Cerebral White Matter | 0.562218 |
| Planum Temporale | 0.561479 |
| Lateral Occipital Cortex, inferior division | 0.561334 |
| Insular Cortex | 0.542884 |
| Inferior Temporal Gyrus, posterior division | 0.54044 |
| Supracalcarine Cortex | 0.501136 |
| Frontal Pole | 0.485161 |
| Heschl's Gyrus | 0.465634 |
| Cingulate Gyrus, posterior division | 0.452712 |
| Occipital Fusiform Gyrus | 0.445274 |
| Frontal Medial Cortex | 0.43761 |
| Lingual Gyrus | 0.424415 |
| Occipital Pole | 0.419279 |
| Inferior Temporal Gyrus, anterior division | 0.411437 |
| Precentral Gyrus | 0.410187 |
| Parahippocampal Gyrus, anterior division | 0.393641 |
| Precuneous Cortex | 0.392317 |
| Superior Temporal Gyrus, anterior division | 0.388624 |
| sub_Right Accumbens | 0.385866 |
| Temporal Fusiform Cortex, anterior division | 0.372217 |
| Temporal Fusiform Cortex, posterior division | 0.356191 |
| sub_Left Putamen | 0.352169 |
| Middle Temporal Gyrus, anterior division | 0.345284 |
| sub_Left Pallidum | 0.343904 |
| sub_Right Pallidum | 0.335488 |
| Superior Frontal Gyrus | 0.269164 |
| Temporal Pole | 0.268235 |
| sub_Right Caudate | 0.205006 |
| **FA features** |  |
| FA_Genu of corpus callosum | 1.958361 |
| FA_Superior fronto-occipital fasciculus R | 1.896877 |
| FA_t_Forceps minor | 1.864198 |
| FA_t_Anterior thalamic radiation R | 1.728985 |
| FA_Superior fronto-occipital fasciculus L | 1.673685 |
| FA_Anterior corona radiata L | 1.614699 |
| FA_Body of corpus callosum | 1.580523 |
| FA_t_Anterior thalamic radiation L | 1.527187 |
| FA_Anterior corona radiata R | 1.511294 |
| FA_Tapetum R | 1.419653 |
| FA_Splenium of corpus callosum | 1.415819 |
| FA_Sagittal stratum R | 1.312461 |
| FA_Posterior thalamic radiation L | 1.292949 |
| FA_t_Inferior fronto-occipital fasciculus R | 1.287675 |
| FA_Anterior limb of internal capsule L | 1.268505 |
| FA_t_Inferior longitudinal fasciculus R | 1.266632 |
| FA_Middle cerebellar peduncle | 1.259746 |
| FA_Cerebral peduncle R | 1.24866 |
| FA_Anterior limb of internal capsule R | 1.223287 |
| FA_t_Inferior fronto-occipital fasciculus L | 1.184462 |
| FA_Posterior thalamic radiation R | 1.1641 |
| FA_t_Forceps major | 1.159534 |
| FA_Cingulum (hippocampus) R | 1.108283 |
| FA_Cerebral peduncle L | 1.043891 |
| FA_t_Cingulum (cingulate gyrus) R | 1.04348 |
| FA_t_Uncinate fasciculus R | 0.949477 |
| FA_Inferior fronto-occipital fasciculus R | 0.942303 |
| FA_t_Cingulum (hippocampus) R | 0.924582 |
| FA_Superior cerebellar peduncle L | 0.844856 |
| FA_Retrolenticular part of internal capsule R | 0.825383 |
| FA_Superior cerebellar peduncle R | 0.798885 |
| FA_Posterior limb of internal capsule R | 0.792781 |
| FA_t_Uncinate fasciculus L | 0.783348 |
| FA_Cingulum (hippocampus) L | 0.77811 |
| FA_Medial lemniscus R | 0.76478 |
| FA_t_Superior longitudinal fasciculus R | 0.761933 |
| FA_Uncinate fasciculus L | 0.754614 |
| FA_t_Superior longitudinal fasciculus L | 0.751305 |
| FA_Superior longitudinal fasciculus R | 0.746655 |
| FA_Posterior corona radiata L | 0.728275 |
| FA_Inferior cerebellar peduncle R | 0.684603 |
| FA_Posterior corona radiata R | 0.659493 |
| FA_t_Cingulum (hippocampus) L | 0.642531 |
| FA_Posterior limb of internal capsule L | 0.623929 |
| FA_Inferior fronto-occipital fasciculus L | 0.622452 |
| FA_Inferior cerebellar peduncle L | 0.601479 |
| FA_t_Superior longitudinal fasciculus (temporal part) L | 0.54742 |
| FA_t_Superior longitudinal fasciculus (temporal part) R | 0.489782 |
| FA_Uncinate fasciculus R | 0.482929 |
| FA_Corticospinal tract R | 0.468444 |
| FA_Superior corona radiata R | 0.454273 |
| FA_External capsule L | 0.442506 |
| FA_Superior longitudinal fasciculus L | 0.430556 |
| FA_t_Corticospinal tract L | 0.413143 |
| FA_Pontine crossing tract | 0.351455 |
| FA_t_Corticospinal tract R | 0.325897 |
| FA_Superior corona radiata L | 0.296811 |
| **MD Features** |  |
| MD_Superior fronto-occipital fasciculus R | -2.00873 |
| MD_Sagittal stratum R | -1.8837 |
| MD_Anterior corona radiata R | -1.74221 |
| MD_t_Inferior fronto-occipital fasciculus L | -1.73813 |
| MD_Anterior corona radiata L | -1.73667 |
| MD_Superior corona radiata R | -1.73183 |
| MD_Posterior corona radiata R | -1.64691 |
| MD_t_Inferior fronto-occipital fasciculus R | -1.62937 |
| MD_Superior fronto-occipital fasciculus L | -1.62915 |
| MD_t_Anterior thalamic radiation L | -1.60114 |
| MD_Fornix (cres) R | -1.58907 |
| MD_Genu of corpus callosum | -1.55255 |
| MD_Body of corpus callosum | -1.53999 |
| MD_Posterior corona radiata L | -1.52617 |
| MD_t_Anterior thalamic radiation R | -1.52524 |
| MD_Retrolenticular part of internal capsule R | -1.52451 |
| MD_Splenium of corpus callosum | -1.4668 |
| MD_Fornix | -1.44386 |
| MD_Anterior limb of internal capsule R | -1.43263 |
| MD_Tapetum R | -1.42402 |
| MD_Superior corona radiata L | -1.41996 |
| MD_Posterior thalamic radiation R | -1.41247 |
| MD_Superior longitudinal fasciculus R | -1.40347 |
| MD_t_Superior longitudinal fasciculus (temporal part) R | -1.35871 |
| MD_Retrolenticular part of internal capsule L | -1.35595 |
| MD_Posterior thalamic radiation L | -1.33523 |
| MD_Fornix (cres) L | -1.32084 |
| MD_Sagittal stratum L | -1.28111 |
| MD_External capsule R | -1.26466 |
| MD_t_Superior longitudinal fasciculus L | -1.24879 |
| MD_t_Inferior longitudinal fasciculus R | -1.18136 |
| MD_t_Superior longitudinal fasciculus R | -1.17447 |
| MD_t_Inferior longitudinal fasciculus L | -1.17202 |
| MD_Anterior limb of internal capsule L | -1.14579 |
| MD_Cingulum (cingulate gyrus) R | -1.11587 |
| MD_t_Superior longitudinal fasciculus (temporal part) L | -1.10262 |
| MD_Cingulum (hippocampus) R | -1.04119 |
| MD_Posterior limb of internal capsule R | -1.03913 |
| MD_t_Corticospinal tract L | -1.03735 |
| MD_Inferior fronto-occipital fasciculus R | -1.03075 |
| MD_t_Cingulum (cingulate gyrus) L | -1.02469 |
| MD_t_Corticospinal tract R | -1.0181 |
| MD_External capsule L | -1.01602 |
| MD_t_Cingulum (hippocampus) R | -0.99886 |
| MD_Superior longitudinal fasciculus L | -0.99175 |
| MD_t_Uncinate fasciculus R | -0.9724 |
| MD_Corticospinal tract L | -0.9345 |
| MD_Cingulum (cingulate gyrus) L | -0.91743 |
| MD_Middle cerebellar peduncle | -0.85267 |
| MD_Cingulum (hippocampus) L | -0.80645 |
| MD_Posterior limb of internal capsule L | -0.80599 |
| MD_t_Cingulum (cingulate gyrus) R | -0.80583 |
| MD_Corticospinal tract R | -0.77641 |
| MD_t_Forceps major | -0.76374 |
| MD_Cerebral peduncle R | -0.74661 |
| MD_Uncinate fasciculus R | -0.69328 |
| MD_Superior cerebellar peduncle R | -0.66798 |
| MD_Superior cerebellar peduncle L | -0.65676 |
| MD_Inferior cerebellar peduncle R | -0.5985 |
| MD_Medial lemniscus R | -0.58574 |
| MD_t_Cingulum (hippocampus) L | -0.52745 |
| MD_Medial lemniscus L | -0.47367 |
| MD_Cerebral peduncle L | -0.47105 |
| MD_Inferior cerebellar peduncle L | -0.19881 |

FA: fraction anisotropy, MD: mean diffusivity, L: left hemisphere, R: right hemisphere, t_: tracts from JHU white-matter tractography atlas.

**TABLE 3** | Significant features between subtype A and NC

| **sMRI volumes** | **P_Value_FDR** |
| --- | --- |
| Cingulate Gyrus, posterior division | 0.200184 |
| Angular Gyrus | 0.200184 |
| Supramarginal Gyrus, posterior division | 0.200184 |
| Postcentral Gyrus | 0.294729 |
| sub_Right Accumbens | 0.294729 |
| sub_Right Pallidum | 0.294729 |
| sub_Right Cerebral Cortex | 0.294729 |
| sub_Left Lateral Ventricle | 0.294729 |
| Middle Frontal Gyrus | 0.302082 |
| Supramarginal Gyrus, anterior division | 0.32218 |
| Frontal Pole | 0.330457 |
| Superior Temporal Gyrus, posterior division | 0.330457 |
| Subcallosal Cortex | 0.355665 |
| Lingual Gyrus | 0.355665 |
| Insular Cortex | 0.359847 |
| sub_Right Amygdala | 0.359847 |
| Paracingulate Gyrus | 0.465352 |
| sub_Right Cerebral White Matter | 0.465352 |
| sub_Left Amygdala | 0.465352 |
| Cingulate Gyrus, anterior division | 0.465352 |
| Frontal Orbital Cortex | 0.465352 |
| Lateral Occipital Cortex, superior division | 0.465352 |
| sub_Left Cerebral White Matter | 0.544023 |
| sub_Left Pallidum | 0.544023 |
| Intracalcarine Cortex | 0.544023 |
| Planum Polare | 0.546594 |
| Frontal Medial Cortex | 0.546594 |
| Planum Temporale | 0.546594 |
| Frontal Operculum Cortex | 0.546594 |
| sub_Left Thalamus | 0.546594 |
| Middle Temporal Gyrus, posterior division | 0.567459 |
| Precuneous Cortex | 0.57041 |
| sub_Left Putamen | 0.57041 |
| sub_Left Caudate | 0.57041 |
| Occipital Pole | 0.57041 |
| Lateral Occipital Cortex, inferior division | 0.59508 |
| Temporal Fusiform Cortex, anterior division | 0.70186 |
| Occipital Fusiform Gyrus | 0.70186 |
| Supracalcarine Cortex | 0.70186 |
| Temporal Fusiform Cortex, posterior division | 0.70186 |
| Inferior Temporal Gyrus, posterior division | 0.70186 |
| Precentral Gyrus | 0.750192 |
| Middle Temporal Gyrus, temporooccipital part | 0.750192 |
| Parahippocampal Gyrus, posterior division | 0.750192 |
| Temporal Pole | 0.750192 |
| Inferior Temporal Gyrus, anterior division | 0.750192 |
| Superior Temporal Gyrus, anterior division | 0.750192 |
| Inferior Frontal Gyrus, pars opercularis | 0.750192 |
| Inferior Temporal Gyrus, temporooccipital part | 0.767562 |
| Central Opercular Cortex | 0.772789 |
| Heschl's Gyrus | 0.847854 |
| sub_Right Caudate | 0.86153 |
| Inferior Frontal Gyrus, pars triangularis | 0.905823 |
| Superior Frontal Gyrus | 0.931738 |
| Parahippocampal Gyrus, anterior division | 0.931738 |
| Middle Temporal Gyrus, anterior division | 0.991604 |
| **FA features** |  |
| FA_Posterior thalamic radiation L | 5.82E-07 |
| FA_t_Inferior fronto-occipital fasciculus L | 2.79E-06 |
| FA_Anterior corona radiata L | 3.29E-06 |
| FA_Superior fronto-occipital fasciculus R | 3.29E-06 |
| FA_Anterior corona radiata R | 8.85E-06 |
| FA_Genu of corpus callosum | 8.85E-06 |
| FA_t_Inferior longitudinal fasciculus R | 8.85E-06 |
| FA_t_Inferior fronto-occipital fasciculus R | 9.22E-06 |
| FA_t_Forceps minor | 1.02E-05 |
| FA_Sagittal stratum R | 1.19E-05 |
| FA_Posterior thalamic radiation R | 2.99E-05 |
| FA_t_Anterior thalamic radiation R | 4.12E-05 |
| FA_Middle cerebellar peduncle | 5.51E-05 |
| FA_Anterior limb of internal capsule R | 8.89E-05 |
| FA_Retrolenticular part of internal capsule R | 0.000208 |
| FA_t_Anterior thalamic radiation L | 0.000228 |
| FA_Anterior limb of internal capsule L | 0.000231 |
| FA_t_Superior longitudinal fasciculus R | 0.000267 |
| FA_Superior longitudinal fasciculus R | 0.000273 |
| FA_Cerebral peduncle R | 0.00034 |
| FA_Cerebral peduncle L | 0.000351 |
| FA_Superior cerebellar peduncle R | 0.000421 |
| FA_Inferior fronto-occipital fasciculus R | 0.000444 |
| FA_Superior fronto-occipital fasciculus L | 0.000506 |
| FA_Splenium of corpus callosum | 0.000562 |
| FA_Posterior limb of internal capsule R | 0.000615 |
| FA_Cingulum (hippocampus) R | 0.000681 |
| FA_t_Cingulum (hippocampus) L | 0.000681 |
| FA_t_Superior longitudinal fasciculus (temporal part) R | 0.000816 |
| FA_Superior longitudinal fasciculus L | 0.000816 |
| FA_t_Superior longitudinal fasciculus (temporal part) L | 0.001295 |
| FA_t_Superior longitudinal fasciculus L | 0.001608 |
| FA_Body of corpus callosum | 0.001775 |
| FA_Superior cerebellar peduncle L | 0.001775 |
| FA_Inferior cerebellar peduncle R | 0.002405 |
| FA_t_Cingulum (hippocampus) R | 0.002959 |
| FA_Superior corona radiata R | 0.003016 |
| FA_t_Uncinate fasciculus L | 0.003904 |
| FA_Posterior limb of internal capsule L | 0.003904 |
| FA_Posterior corona radiata L | 0.004499 |
| FA_Superior corona radiata L | 0.006274 |
| FA_t_Corticospinal tract L | 0.006801 |
| FA_Inferior fronto-occipital fasciculus L | 0.007734 |
| FA_t_Corticospinal tract R | 0.007734 |
| FA_t_Forceps major | 0.009837 |
| FA_t_Cingulum (cingulate gyrus) R | 0.011607 |
| FA_Corticospinal tract R | 0.011607 |
| FA_Posterior corona radiata R | 0.011607 |
| FA_t_Uncinate fasciculus R | 0.014229 |
| FA_Cingulum (hippocampus) L | 0.019849 |
| FA_Tapetum R | 0.020991 |
| FA_Inferior cerebellar peduncle L | 0.02321 |
| FA_Uncinate fasciculus R | 0.026319 |
| FA_External capsule L | 0.034968 |
| FA_Medial lemniscus R | 0.089676 |
| FA_Uncinate fasciculus L | 0.390225 |
| FA_Pontine crossing tract | 0.475925 |
| **MD Features** |  |
| MD_t_Inferior fronto-occipital fasciculus L | 0.000539 |
| MD_Superior fronto-occipital fasciculus R | 0.000539 |
| MD_Superior longitudinal fasciculus R | 0.000539 |
| MD_t_Superior longitudinal fasciculus (temporal part) R | 0.000539 |
| MD_Sagittal stratum R | 0.000585 |
| MD_Cingulum (cingulate gyrus) L | 0.000606 |
| MD_Anterior corona radiata L | 0.000682 |
| MD_Superior corona radiata R | 0.000682 |
| MD_t_Inferior fronto-occipital fasciculus R | 0.000682 |
| MD_t_Cingulum (cingulate gyrus) L | 0.000682 |
| MD_Superior longitudinal fasciculus L | 0.000682 |
| MD_t_Superior longitudinal fasciculus (temporal part) L | 0.00074 |
| MD_Superior fronto-occipital fasciculus L | 0.00092 |
| MD_Posterior thalamic radiation R | 0.001031 |
| MD_Anterior corona radiata R | 0.001127 |
| MD_Retrolenticular part of internal capsule R | 0.001127 |
| MD_Body of corpus callosum | 0.001127 |
| MD_Corticospinal tract L | 0.001127 |
| MD_t_Superior longitudinal fasciculus R | 0.001127 |
| MD_Anterior limb of internal capsule R | 0.001127 |
| MD_Superior corona radiata L | 0.001127 |
| MD_Posterior thalamic radiation L | 0.001965 |
| MD_Posterior corona radiata R | 0.002058 |
| MD_Genu of corpus callosum | 0.002182 |
| MD_Posterior corona radiata L | 0.002182 |
| MD_t_Inferior longitudinal fasciculus L | 0.002182 |
| MD_External capsule R | 0.002325 |
| MD_Retrolenticular part of internal capsule L | 0.002663 |
| MD_Splenium of corpus callosum | 0.002751 |
| MD_Corticospinal tract R | 0.003204 |
| MD_t_Corticospinal tract R | 0.003843 |
| MD_Posterior limb of internal capsule R | 0.003843 |
| MD_Sagittal stratum L | 0.005181 |
| MD_Cingulum (hippocampus) R | 0.005569 |
| MD_t_Corticospinal tract L | 0.005977 |
| MD_t_Inferior longitudinal fasciculus R | 0.007627 |
| MD_Fornix (cres) L | 0.008392 |
| MD_Fornix (cres) R | 0.010187 |
| MD_Tapetum R | 0.015338 |
| MD_t_Superior longitudinal fasciculus L | 0.015338 |
| MD_t_Anterior thalamic radiation L | 0.016952 |
| MD_Anterior limb of internal capsule L | 0.018618 |
| MD_Inferior cerebellar peduncle R | 0.018985 |
| MD_t_Anterior thalamic radiation R | 0.020266 |
| MD_Cingulum (cingulate gyrus) R | 0.022912 |
| MD_Inferior fronto-occipital fasciculus R | 0.028738 |
| MD_t_Cingulum (hippocampus) R | 0.031337 |
| MD_t_Cingulum (hippocampus) L | 0.0353 |
| MD_Posterior limb of internal capsule L | 0.0353 |
| MD_t_Cingulum (cingulate gyrus) R | 0.036846 |
| MD_Medial lemniscus L | 0.038548 |
| MD_Superior cerebellar peduncle L | 0.045393 |
| MD_Superior cerebellar peduncle R | 0.048618 |
| MD_Fornix | 0.060559 |
| MD_Cerebral peduncle R | 0.064112 |
| MD_External capsule L | 0.064112 |
| MD_Medial lemniscus R | 0.079241 |
| MD_Uncinate fasciculus R | 0.090238 |
| MD_t_Uncinate fasciculus R | 0.111063 |
| MD_Cingulum (hippocampus) L | 0.134677 |
| MD_Inferior cerebellar peduncle L | 0.146538 |
| MD_t_Forceps major | 0.172268 |
| MD_Middle cerebellar peduncle | 0.172268 |
| MD_Cerebral peduncle L | 0.237949 |

FA: fraction anisotropy, MD: mean diffusivity, L: left hemisphere, R: right hemisphere, t_: tracts from JHU white-matter tractography atlas.

**TABLE 4** | Significant features between subtype B and NC

| **sMRI Features** | **P_vlaue_FDR** |
| --- | --- |
| sub_Left Lateral Ventricle | 0.064641 |
| Central Opercular Cortex | 0.064641 |
| Frontal Operculum Cortex | 0.064641 |
| Inferior Temporal Gyrus, posterior division | 0.064641 |
| Middle Temporal Gyrus, temporooccipital part | 0.064641 |
| Inferior Frontal Gyrus, pars triangularis | 0.064641 |
| Parahippocampal Gyrus, posterior division | 0.064641 |
| Intracalcarine Cortex | 0.064641 |
| Inferior Frontal Gyrus, pars opercularis | 0.064696 |
| Inferior Temporal Gyrus, temporooccipital part | 0.087747 |
| Paracingulate Gyrus | 0.093119 |
| sub_Right Cerebral Cortex | 0.093119 |
| sub_Right Cerebral White Matter | 0.093119 |
| Occipital Fusiform Gyrus | 0.093119 |
| sub_Left Cerebral White Matter | 0.093119 |
| Lateral Occipital Cortex, superior division | 0.106461 |
| Temporal Fusiform Cortex, posterior division | 0.121009 |
| Supracalcarine Cortex | 0.139096 |
| sub_Left Thalamus | 0.14124 |
| sub_Left Amygdala | 0.14124 |
| Subcallosal Cortex | 0.14124 |
| Lateral Occipital Cortex, inferior division | 0.143074 |
| Occipital Pole | 0.170254 |
| Frontal Orbital Cortex | 0.175583 |
| sub_Left Caudate | 0.214177 |
| Middle Temporal Gyrus, posterior division | 0.231473 |
| Middle Frontal Gyrus | 0.231473 |
| sub_Right Amygdala | 0.252657 |
| Cingulate Gyrus, anterior division | 0.252657 |
| Middle Temporal Gyrus, anterior division | 0.252657 |
| Inferior Temporal Gyrus, anterior division | 0.262052 |
| Angular Gyrus | 0.32273 |
| Precuneous Cortex | 0.32273 |
| Precentral Gyrus | 0.32273 |
| Frontal Medial Cortex | 0.32273 |
| Parahippocampal Gyrus, anterior division | 0.371192 |
| Heschl's Gyrus | 0.392901 |
| Planum Polare | 0.469808 |
| Planum Temporale | 0.469808 |
| Lingual Gyrus | 0.515496 |
| Frontal Pole | 0.59348 |
| Temporal Fusiform Cortex, anterior division | 0.599611 |
| Superior Temporal Gyrus, anterior division | 0.607514 |
| sub_Right Caudate | 0.619763 |
| Postcentral Gyrus | 0.743908 |
| Supramarginal Gyrus, posterior division | 0.771076 |
| sub_Right Pallidum | 0.841451 |
| sub_Left Pallidum | 0.841451 |
| Superior Frontal Gyrus | 0.841451 |
| sub_Left Putamen | 0.841451 |
| Supramarginal Gyrus, anterior division | 0.841451 |
| Cingulate Gyrus, posterior division | 0.954246 |
| sub_Right Accumbens | 0.954246 |
| Insular Cortex | 0.954246 |
| Temporal Pole | 0.954246 |
| Superior Temporal Gyrus, posterior division | 0.954246 |
| **FA features** |  |
| FA_Tapetum R | 0.039006 |
| FA_Genu of corpus callosum | 0.039006 |
| FA_Superior fronto-occipital fasciculus L | 0.039006 |
| FA_t_Anterior thalamic radiation R | 0.039006 |
| FA_Body of corpus callosum | 0.046203 |
| FA_t_Anterior thalamic radiation L | 0.05066 |
| FA_t_Forceps minor | 0.050828 |
| FA_Superior fronto-occipital fasciculus R | 0.062958 |
| FA_t_Cingulum (cingulate gyrus) R | 0.062958 |
| FA_t_Forceps major | 0.062958 |
| FA_Uncinate fasciculus L | 0.062958 |
| FA_Splenium of corpus callosum | 0.085234 |
| FA_Anterior limb of internal capsule L | 0.085234 |
| FA_Middle cerebellar peduncle | 0.216386 |
| FA_Anterior corona radiata R | 0.312934 |
| FA_Cerebral peduncle R | 0.312934 |
| FA_Anterior limb of internal capsule R | 0.329945 |
| FA_Medial lemniscus R | 0.427578 |
| FA_Anterior corona radiata L | 0.427578 |
| FA_Sagittal stratum R | 0.427578 |
| FA_Cingulum (hippocampus) R | 0.485554 |
| FA_Cingulum (hippocampus) L | 0.51591 |
| FA_Superior cerebellar peduncle L | 0.51591 |
| FA_Cerebral peduncle L | 0.630078 |
| FA_t_Inferior longitudinal fasciculus R | 0.665514 |
| FA_t_Uncinate fasciculus L | 0.665514 |
| FA_Posterior corona radiata R | 0.665514 |
| FA_t_Cingulum (hippocampus) L | 0.665514 |
| FA_Posterior limb of internal capsule L | 0.665514 |
| FA_Superior cerebellar peduncle R | 0.665514 |
| FA_t_Uncinate fasciculus R | 0.70297 |
| FA_t_Corticospinal tract R | 0.70405 |
| FA_Superior corona radiata R | 0.70405 |
| FA_Posterior thalamic radiation R | 0.70405 |
| FA_Pontine crossing tract | 0.70405 |
| FA_Posterior limb of internal capsule R | 0.70405 |
| FA_Superior longitudinal fasciculus L | 0.705989 |
| FA_t_Cingulum (hippocampus) R | 0.705989 |
| FA_Inferior cerebellar peduncle L | 0.71999 |
| FA_Superior corona radiata L | 0.746346 |
| FA_t_Inferior fronto-occipital fasciculus R | 0.746346 |
| FA_t_Superior longitudinal fasciculus (temporal part) R | 0.76354 |
| FA_Posterior corona radiata L | 0.771478 |
| FA_Inferior fronto-occipital fasciculus L | 0.771478 |
| FA_t_Superior longitudinal fasciculus L | 0.771478 |
| FA_Posterior thalamic radiation L | 0.774562 |
| FA_External capsule L | 0.774562 |
| FA_Uncinate fasciculus R | 0.774562 |
| FA_t_Corticospinal tract L | 0.787221 |
| FA_Inferior cerebellar peduncle R | 0.838056 |
| FA_Corticospinal tract R | 0.838056 |
| FA_Retrolenticular part of internal capsule R | 0.838056 |
| FA_t_Superior longitudinal fasciculus R | 0.922931 |
| FA_t_Inferior fronto-occipital fasciculus L | 0.963103 |
| FA_Superior longitudinal fasciculus R | 0.970539 |
| FA_t_Superior longitudinal fasciculus (temporal part) L | 0.994479 |
| FA_Inferior fronto-occipital fasciculus R | 0.997658 |
| **MD Features** |  |
| MD_t_Anterior thalamic radiation L | 0.004864 |
| MD_t_Anterior thalamic radiation R | 0.004864 |
| MD_Superior fronto-occipital fasciculus L | 0.004864 |
| MD_Superior fronto-occipital fasciculus R | 0.006 |
| MD_Sagittal stratum R | 0.009131 |
| MD_Anterior limb of internal capsule L | 0.009713 |
| MD_Anterior corona radiata R | 0.011649 |
| MD_Superior corona radiata R | 0.011649 |
| MD_Superior corona radiata L | 0.011649 |
| MD_Posterior corona radiata R | 0.01331 |
| MD_Cingulum (cingulate gyrus) R | 0.01331 |
| MD_Splenium of corpus callosum | 0.01331 |
| MD_Fornix | 0.013845 |
| MD_Tapetum R | 0.013845 |
| MD_t_Superior longitudinal fasciculus L | 0.013845 |
| MD_Posterior thalamic radiation R | 0.013845 |
| MD_Genu of corpus callosum | 0.014445 |
| MD_Fornix (cres) L | 0.014445 |
| MD_t_Inferior longitudinal fasciculus R | 0.014445 |
| MD_Middle cerebellar peduncle | 0.014445 |
| MD_t_Forceps major | 0.014445 |
| MD_Sagittal stratum L | 0.014496 |
| MD_External capsule L | 0.014916 |
| MD_Anterior corona radiata L | 0.016019 |
| MD_Anterior limb of internal capsule R | 0.017956 |
| MD_Posterior thalamic radiation L | 0.017956 |
| MD_Body of corpus callosum | 0.018552 |
| MD_Fornix (cres) R | 0.018628 |
| MD_Posterior corona radiata L | 0.025337 |
| MD_t_Inferior fronto-occipital fasciculus R | 0.031064 |
| MD_t_Inferior fronto-occipital fasciculus L | 0.037357 |
| MD_Posterior limb of internal capsule L | 0.037357 |
| MD_t_Superior longitudinal fasciculus R | 0.037596 |
| MD_Cingulum (hippocampus) L | 0.038909 |
| MD_t_Inferior longitudinal fasciculus L | 0.038996 |
| MD_Retrolenticular part of internal capsule L | 0.042058 |
| MD_Retrolenticular part of internal capsule R | 0.049656 |
| MD_External capsule R | 0.059147 |
| MD_t_Corticospinal tract L | 0.064568 |
| MD_Superior longitudinal fasciculus R | 0.064568 |
| MD_t_Superior longitudinal fasciculus (temporal part) R | 0.065584 |
| MD_Superior cerebellar peduncle L | 0.065584 |
| MD_Superior cerebellar peduncle R | 0.074756 |
| MD_t_Superior longitudinal fasciculus (temporal part) L | 0.076241 |
| MD_t_Cingulum (cingulate gyrus) R | 0.082153 |
| MD_t_Uncinate fasciculus R | 0.108993 |
| MD_Posterior limb of internal capsule R | 0.108993 |
| MD_t_Corticospinal tract R | 0.124654 |
| MD_Cerebral peduncle R | 0.130153 |
| MD_t_Cingulum (cingulate gyrus) L | 0.168805 |
| MD_Cingulum (hippocampus) R | 0.186628 |
| MD_Superior longitudinal fasciculus L | 0.226179 |
| MD_t_Cingulum (hippocampus) R | 0.238691 |
| MD_Cingulum (cingulate gyrus) L | 0.239386 |
| MD_Inferior fronto-occipital fasciculus R | 0.256814 |
| MD_Uncinate fasciculus R | 0.256814 |
| MD_Corticospinal tract L | 0.309096 |
| MD_Cerebral peduncle L | 0.31217 |
| MD_t_Cingulum (hippocampus) L | 0.495466 |
| MD_Corticospinal tract R | 0.547644 |
| MD_Medial lemniscus R | 0.548947 |
| MD_Inferior cerebellar peduncle R | 0.591036 |
| MD_Medial lemniscus L | 0.716784 |
| MD_Inferior cerebellar peduncle L | 0.716784 |

FA: fraction anisotropy, MD: mean diffusivity, L: left hemisphere, R: right hemisphere, t_: tracts from JHU white-matter tractography atlas.

**TABLE 5** | Features selected for correlation analysis

| **sMRI features** |
| --- |
| sub_Left Lateral Ventricle |
| Angular Gyrus |
| Frontal Operculum Cortex |
| Central Opercular Cortex |
| Paracingulate Gyrus |
| sub_Right Amygdala |
| Middle Temporal Gyrus, temporooccipital part |
| sub_Left Amygdala |
| sub_Left Thalamus |
| Lateral Occipital Cortex, superior division |
| **FA features** |
| FA_Genu of corpus callosum |
| FA_Superior fronto-occipital fasciculus R |
| FA_t_Forceps minor |
| FA_t_Anterior thalamic radiation R |
| FA_Superior fronto-occipital fasciculus L |
| FA_Anterior corona radiata L |
| FA_Body of corpus callosum |
| FA_t_Anterior thalamic radiation L |
| FA_Anterior corona radiata R |
| FA_Tapetum R |
| FA_Posterior thalamic radiation L |
| FA_t_Inferior fronto-occipital fasciculus L |
| FA_t_Inferior fronto-occipital fasciculus R |
| FA_t_Inferior longitudinal fasciculus R |
| FA_Sagittal stratum R |
| **MD features** |
| MD_Superior fronto-occipital fasciculus R |
| MD_Sagittal stratum R |
| MD_Anterior corona radiata R |
| MD_t_Inferior fronto-occipital fasciculus L |
| MD_Anterior corona radiata L |
| MD_Superior corona radiata R |
| MD_Posterior corona radiata R |
| MD_t_Inferior fronto-occipital fasciculus R |
| MD_Superior fronto-occipital fasciculus L |
| MD_t_Anterior thalamic radiation L |
| MD_Superior longitudinal fasciculus R |
| MD_t_Superior longitudinal fasciculus (temporal part) R |
| MD_Cingulum (cingulate gyrus) L |
| MD_t_Anterior thalamic radiation R |
| MD_Fornix |
| MD_Tapetum R |
| MD_Anterior limb of internal capsule L |

FA: fraction anisotropy, MD: mean diffusivity, L: left hemisphere, R: right hemisphere, t_: tracts from JHU white-matter tractography atlas.
